# Variants of the *X*-phase in the Mn–Co–Ge system

**DOI:** 10.1107/S2053229621002370

**Published:** 2021-03-09

**Authors:** Vitalii Shtender, Simon R. Larsen, Martin Sahlberg

**Affiliations:** a Department of Chemistry, Ångström Laboratory, Uppsala University, Box 538, 75121 Uppsala, Sweden

**Keywords:** inter­metallic com­pound, crystal structure, *X*-phase, Mn-Co-Ge system, manganese, cobalt, germanide

## Abstract

We report two new variants of the *X*-phase (ortho­rhom­bic, space group *Pnnm*) derived from the Mn–Co–Ge system. Two com­positionally related crystals were investigated by means of single-crystal X-ray diffraction (XRD) and energy dispersive spectroscopy (EDS). The Mn_14.9_Co_15.5_Ge_6.6_ and Mn_14_Co_16.2_Ge_6.8_ inter­metallic com­pounds are part of the homogeneity region of the *X*-phase and adopt the Mn_14_(Mn_0.11_Co_0.64_Si_0.25_)_23_ structure type. The com­position obtained from refinement of the XRD data is in agreement with the EDS results. In the present study, chemical disorder was only detected on the 8*h* positions. The ordering is com­pared with other members of the *X*-phase family and shows that the degree of disordering depends on the chemical com­position. No com­pletely ordered variants of the *X*-phase have yet been reported.

## Abstract 2

The ordering of two new variants of the *X*-phase derived from the Mn–Co–Ge system is com­pared with other members of the *X*-phase family and shows that the degree of disordering depends on the chemical com­position. No com­pletely ordered variants of the *X*-phase have yet been reported.

## Introduction   

Topologically close packed (TCP) phases or Frank–Kasper phases as they are also known encom­pass a large number of inter­metallic com­pounds (Dshemuchadse & Steurer, 2015 ▸; Ovchinnikov *et al.*, 2020 ▸). As a result, TCP phases often appear in various widely used alloys such as steels. In single-crystal superalloys they are reported to reduce microstructural stability, promote creep porosities and induce cracking and crack propagation (Tan *et al.*, 2020 ▸). In high-entropy alloys, they often appear in the form of the σ-phase and have been reported to improve strength while providing good ductility (Jo *et al.*, 2018 ▸). Due to this, control of the formation of these phases is desired as they have a large impact on the mech­anical properties of materials.

Conventional close packing of atoms of the same size create tetra­hedral and octa­hedral inter­stitial holes. When atoms of slightly different sizes pack together, TCPs can arise as they achieve a better packing by forming small non-uniform tetra­hedral inter­stices. The non-uniform nature of the tetra­hedral inter­stices allow for coordination numbers of 12, 14, 15 and 16 (Frank & Kasper, 1958 ▸; Wang & Mar, 2001 ▸; Ovchinnikov *et al.*, 2020 ▸). The Valence Electron Concentration (VEC) also plays a role in determining the structure and stability of the TCP phase, so much so that maps based on the VEC and divergence from the average atomic size can be used to predict the various phases seen (Seiser *et al.*, 2011 ▸; Hammerschmidt *et al.*, 2013 ▸). Theoretical calculations also indicate that magnetism has a minor effect on the structural stability of certain phases (Hammerschmidt *et al.*, 2013 ▸).

The *X*-phase is a rare structure type that has only been reported for two systems. The Mn–Co–Si system, from which the base structure is derived, was discovered in the 1970s by two independent groups (Yarmolyuk *et al.*, 1970 ▸; Manor *et al.*, 1972 ▸). This structure has been reported as Mn_15.84_Co_15.87_Si_5.29_ and Mn_44.4_Co_40.0_Si_15.1_, respectively. In general, these phases are assigned to the Mn_14_(Mn_0.11_Co_0.64_Si_0.25_)_23_ structure type (Villars & Cenzual, 2016 ▸), where seven independent positions relate to Mn and the other nine positions are mixed Co/Mn or Si/Co/Mn. Investigation of the phase diagram of the ternary Mn–Co–Si system at 800 °C revealed the formation of Mn_16.5_Co_14.8_Si_5.7_ (Kuz’ma & Gladyshevskii, 1964 ▸), while at 1000 °C, the authors reported Mn_16.5_Co_14.8_Si_5.7_, as well as Mn_3_Co_3_Si (*Y*-phase) (Bardos *et al.*, 1966 ▸). A rough approximation for the stoichiometry of the *X*-phase can be given close to 3–3–1, as also used for the *Y*-phase (Gupta, 2006 ▸). There has been some discussion as to whether the structure is instead the *Y*-phase (Manor *et al.*, 1972 ▸; Gupta, 2006 ▸); the similarity of the diffraction patterns indicates that they might be the same phase with a large homogeneity region. The second system reported in 2001 is com­posed of Nb–Ni–Sb (Wang & Mar, 2001 ▸) and was reported as the Nb_28_Ni_33.5_Sb_12.5_ [Nb_14_Ni_16.75_Sb_6.25_ or Nb_14_(Ni_0.728_Sb_0.272_)_23_] ternary com­pound, which crystalized in the *X*-phase structure type.

In this article, the syntheses and crystal structures of two new Mn–Co–Ge com­pounds, representatives of the *X*-phase, will be discussed.

## Experimental   

### Synthesis   

This study was initiated based on separate results obtained during the investigation of Mn_2_Co_3_Ge, a com­pound which was selected as a permanent magnet candidate (Vishina *et al.*, 2021 ▸). Initial trials revealed the magnetic Heusler phase MnCo_2_Ge (Buschow *et al.*, 1983 ▸) as being the main com­peting phase in that region of the phase diagram. The synthesis of the Mn_2_Co_3_Ge com­pound was achieved by arc melting and negligible loses of Mn (1–3 wt%) were detected in most cases. In the event of larger losses, more Mn was added to com­pensate for the losses and to avoid the formation of MnCo_2_Ge. It was found that an additional 3 wt% of Mn decreased the amount of MnCo_2_Ge impurities. Based on this, Mn_2_Co_3_Ge alloys with 5, 7 and 10 wt% of excess Mn were prepared. However, while it did reduce the amount of MnCo_2_Ge, in most cases, it did not result in higher purity of the sought-after phase. The samples of 7 and 10 wt% excess Mn did instead reveal the existence of a new phase, the crystal structure of which is presented herein.

Samples of Mn_2_Co_3_Ge+7%Mn and Mn_2_Co_3_Ge+10%Mn were synthesized by arc melting Co (99.9+%, Alfa Aesar), Mn (99.7%, Höganäs) and Ge (99.999%, Kurt J. Lesker) under an argon atmosphere. A titanium getter was used to reduce oxygen contamination and the samples were flipped and remelted three times to promote homogeneity. Samples were placed in Al_2_O_3_ crucibles and then sealed in evacuated quartz tubes for annealing. Heat treatment was carried out for 7 d at 800 °C, after which samples were quenched with water.

### Characterization   

Crystals were picked up from the Mn–Co–Ge alloys by fragmentation and analyzed. A Bruker D8 single-crystal X-ray diffractometer with Mo *K*α radiation (λ = 0.71073 Å) up­graded with an Incoatec Microfocus Source (IµS, beam size ∼100 µm at the sample position) and an APEXII CCD area detector (6 × 6 cm) was used to collect single-crystal X-ray diffraction (SCXRD) intensities at room temperature. The final cycle of refinement was carried out anisotropically for all species converging with low residuals and a flat difference Fourier map. The atomic positions were standardized with the use of the program *STRUCTURE TIDY* implemented in *PLATON* (Spek, 2020 ▸).

Additional methods, such as scanning electron microscopy (SEM) on a Zeiss Merlin SEM instrument equipped with a secondary electron (SE) detector and an energy-dispersive X-ray spectrometer (EDS), were employed to confirm the com­position of the title com­pounds. The samples used for electron microscopy analysis were prepared by standard metallographic techniques through grinding with SiC paper. For the final polishing, a mixture of SiO_2_ and H_2_O was used. Furthermore, the sample purity was checked by means of powder X-ray diffraction (PXRD) on a Bruker D8 X-ray diffractometer with a Lynx-eye position-sensitive detector and Cu *K*α radiation on a zero-background single-crystal Si sample holder. Phase analysis of the X-ray data using the Rietveld method was carried out with *FULLPROF* software (Rodriguez-Carvajal, 2001 ▸).

The crystal structures of the new *X*-phase representatives were solved by single-crystal X-ray diffraction data analysis using the procedures described above. PXRD phase analysis showed that Mn_2_Co_3_Ge+7%Mn was a multiphase alloy, while Mn_2_Co_3_Ge+10%Mn consisted of a single phase. The elemental com­position obtained from refinement of the crystal structure data was found to be Mn_40.2_Co_41.9_Ge_17.9_ and Mn_37.8_Co_43.9_Ge_18.3_ for the two crystals, respectively, agreeing with EDS results [Mn_40.4 (5)_Co_42.0 (7)_Ge_17.6 (3)_ and Mn_37.7 (9)_Co_45.1 (9)_Ge_17.2 (5)_]. Tables 1 ▸ and 2 ▸ present crystallographic data and experimental details for Mn_14.89 (5)_Co_15.48 (4)_Ge_6.62 (2)_ and Mn_14_Co_16.16 (3)_Ge_6.84 (3)_. Anisotropic displacement parameters, inter­atomic distances and angles are provided as supporting information. For simplicity, the limits of com­position in the text will be referred to as Mn_14.9_Co_15.5_Ge_6.6_ and Mn_14_Co_16.2_Ge_6.8_.

## Results and discussion   

Detailed crystal structure chemistry for the previously studied representatives of the Mn_14_(Mn_0.11_Co_0.64_Si_0.25_)_23_ structure type are presented elsewhere (Yarmolyuk *et al.*, 1970 ▸; Manor *et al.*, 1972 ▸; Wang & Mar, 2001 ▸). In this work, two crystals of similar com­position were studied with the aim of com­paring the ordering/disordering of the atoms in the structure. Two ternary inter­metallic com­pounds with ortho­rhom­bic structures (space group *Pnnm*) and very negligible changes in the unit-cell parameters indicated a small homogeneity region of the *X*-phase which was further supported by EDS analysis. The Ge content is mostly stable, while the majority of changes occur along the Mn/Co line. For both com­positions, it was established that Mn occupies seven independent 4*g* positions. Three Co and two Ge atoms also occupy independent 4*g* positions, with the final Ge atom occupying the position at 2*a*. This holds true for the Mn_14.9_Co_15.5_Ge_6.6_ and Mn_14_Co_16.2_Ge_6.8_ com­positions.

**Figure 1:**
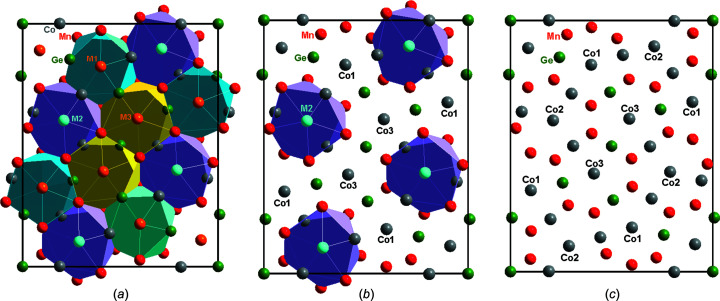
Schematic presentation of the disordering in the 8*h* positions with respect to the chemical com­positions for (*a*) disordered Mn_14.9_Co_15.5_Ge_6.6_, (*b*) partially disordered Mn_14_Co_16.2_Ge_6.8_ and (*c*) hypothetically ordered ‘Mn_14_Co_18_Ge_5_’. All projections of the ortho­rhom­bic unit cells are presented on the *ba* plane. Atoms names are as used in Table 2 ▸.

Differences are present only at the 8*h* positions which are of higher multiplicity. The studied com­pounds have a large degree of Co/Ge inter­mixing on the 8*h* position of *M*2 (see Table 2 ▸). This is likely due to the nearest neighbours of *M*2 (Co/Ge) consisting exclusively of Mn and Co and not Ge [in Fig. 1 ▸, the outlined icosa­hedra (CN = 12) for *M*2 are Mn_7_Co_3_ *M*2_2_]. No clear Ge—Ge bonds that could relate to the sum of atomic radii (*r*_Ge_ = 1.22 Å; Pearson, 1972 ▸) could be discerned in this structure. It can only be realized in the case of *M*2, for which two other *M*2 are as close as 2.3826 (4) and 2.4548 (4) Å. One of these distances could be regarded as the Ge—Ge, Co—Ge or Co—Co inter­atomic distance since atomic radii of Co and Ge are similar (*r*_Co_ = 1.25 and *r*_Ge_ = 1.22 Å; Pearson, 1972 ▸). It should be noted that for other com­pounds of the Mn–Co–Ge and Co–Ge systems, it is common to have shorter Ge—Ge, Co—Ge or Co—Co inter­atomic distances than the sum of the atomic radii (Villars & Cenzual, 2016 ▸).

The last two 8*h* positions (*M*1 and *M*3) are a statistical mixture of Mn and Co for Mn_14.9_Co_15.5_Ge_6.6_, while for Mn_14_Co_16.2_Ge_6.8_, they are occupied solely by Co. As the latter is Mn-lean this makes sense. The Mn/Co occupational ratio at the *M*1 site was refined and the refinement remained stable, while the ratio at *M*3 had to be constrained due to instability of the refinement. This procedure was deemed applicable as the Fourier map and *R* factors improved and the calculated com­position corresponded well to results attained from EDS measurements. The unstable Mn/Co occupational ratio at *M*3 cannot be explained by the smaller volume of the surrounding icosa­hedron and thus the larger electron density. The volume of the *M*3-related icosa­hedron is 45.6 Å^3^, while for *M*1 it is only 43.8 Å^3^. An alternative explanation can be sought by examining the ligands for each central *Mx* atom. As was mentioned, the *M*2 site only has Mn and Co atoms surrounding it, while *M*1 has two atoms of Ge present and *M*3 has three Ge atoms (*M*1@Mn_6_Co_2_ *M*1_2_Ge_2_ and *M*3@Mn_6_Co_1_ *M*3_2_Ge_3_). Polyhedra for *M*3 are always in pairs (while others alternate), sharing one of the Ge atoms between them. Considering that Ge is the most electronegative atom in the present case [χ_Ge_= 2.01, χ_Co_= 1.88 and χ_Mn_= 1.55, according to the Pauling scale (Pauling, 1932 ▸)] and that the *M*3—Ge distances [2.3543 (3), 2.3942 (3) and 2.3948 (2) Å] are the shortest in the structure [for com­parison, the *M*2—*M*2 distance is 2.3826 (4) Å], this might be a reason why the position of *M*3 becomes unstable with the introduction of Mn (*r*_Mn_ = 1.27 Å; Pearson, 1972 ▸). The Co—Ge distances around *M*3 are in the range 2.3911 (4)–2.4564 (4) Å, which is similar to what is seen for *M*2, but the shortest Mn—Ge distance of 2.7233 (5) Å in the same area exhibits a sizeable difference [Mn—*M*2 = 2.6607 (4) Å]. Also, Ge lacks at least one electron in the *p*-orbital to be half-filled (4*p* ^2^), while Co has its excess at *d* (3*d* ^7^4*s* ^2^, *d*-orbital more than half-filled) and Mn is stable in the *d*-orbital (3*d* ^5^4*s* ^2^, *d*-orbital half-filled). By introducing Mn with a larger atomic radius than Co we decrease the distance of the central atom to Ge and remove the unpaired electrons of Co from the area near Ge. For this reason, the statistical mixture of Mn/Co in *M*3, unlike *M*1, cannot be stable during refinement. To summarize, though many different trials of the refinements were carried out, the presented results were found to be the best statistically that also agreed with EDS results. Nevertheless, the presented arguments are our way of explaining the outlined problem at the *M*3 site. It is difficult to conclude whether it is refinement instability or chemical/structural instability from the available data. Our results do not allow com­pletely separate Mn and Co since this is not discernible with XRD (difference of only two electrons) and to differentiate between them neutrons are needed.

The studied com­pounds (Mn_14.9_Co_15.5_Ge_6.6_ and Mn_14_Co_16.2_Ge_6.8_) are com­positionally related to each other and the other members [Mn_15.84_Co_15.87_Si_5.29_ (Yarmolyuk *et al.*, 1970 ▸), Mn_16.46_Co_14.80_Si_5.75_ (Manor *et al.*, 1972 ▸) and Nb_14_Ni_16.78_Sb_6.22_ (Wang & Mar, 2001 ▸)] of the *X*-phase with the Mn_14_(Mn_0.11_Co_0.64_Si_0.25_)_23_ structure type. In all cases, the Mn atoms occupied seven independent positions (in the case of Nb_14_Ni_16.78_Sb_6.22_, the Nb atoms occupy the same positions instead). For the first Si-based com­pound, the other positions were occupied by mixed Mn/Co/Si atoms and the *z* parameters at the 8*h* positions were fixed (Yarmolyuk *et al.*, 1970 ▸). The second Si-based com­pound had a slightly higher degree of ordering, where four positions were shared between Mn/Co/Si and five other positions were only shared between Mn/Co (*z* at the 8*h* positions were refined) (Manor *et al.*, 1972 ▸). That inter­mixing is seen for all elements on so many positions is likely related to the lack of the high-quality data, as the studies were carried out in the 1970s. Contrary to those studies, the most recent publication on Nb_14_Ni_16.78_Sb_6.22_ (Wang & Mar, 2001 ▸) presents a very detailed refinement procedure sup­ported by extended Hückel band structure calculations. In terms of numbers of elements and structural features, Nb_14_Ni_16.78_Sb_6.22_ relates closely to the presented Mn_14_Co_16.2_Ge_6.8_ com­pound. The inter­mixing of Ni/Sb on one 8*h* position is similar to the inter­mixing of Co/Ge presented here. Minor differences are seen on the 4*g* positions where Ni/Sb was found to inter­mix as well, while only Ge was seen to be present here. This could relate to the difference in the homogeneity regions or the nature of the elements. The same might be applicable for the Si-based com­pounds, but a detailed analysis of these old com­pounds would be needed to confirm this. The currently known *X*-phases are all of the same structure type, with the minor differences of the ordering/disordering at some crystallographic positions being a key differentiator.

## Supplementary Material

Crystal structure: contains datablock(s) Mn14Co16.2Ge6.8, Mn14.9Co15.5Ge6.6, global. DOI: 10.1107/S2053229621002370/ef3015sup1.cif

Structure factors: contains datablock(s) Mn14Co16.2Ge6.8. DOI: 10.1107/S2053229621002370/ef3015Mn14Co16.2Ge6.8sup2.hkl

Structure factors: contains datablock(s) Mn14.9Co15.5Ge6.6. DOI: 10.1107/S2053229621002370/ef3015Mn14.9Co15.5Ge6.6sup3.hkl

Supporting information file. DOI: 10.1107/S2053229621002370/ef3015sup4.pdf

CCDC references: 2057512, 2057511

## Figures and Tables

**Table 1:** Crystallographic data and structure refinement parameters for the studied Mn–Co–Ge single crystals Experiments were carried out at 296 K with Mo *K*α radiation using a Bruker APEXII CCD diffractometer (see *Characterization*, §2.2). The absorption correction was mpirical (using intensity measurements) (*SADABS*; Bruker, 2015 ▸).

	**Mn_14.9_Co_15.5_Ge_6.6_**	**Mn_14_Co_16.2_Ge_6.8_**
Compound	Mn_14.89 (5)_Co_15.48 (4)_Ge_6.62 (2)_	Mn_14_Co_16.16 (3)_Ge_6.84 (3)_
Summary formula	Mn_29.79 (10)_Co_30.97 (8)_Ge_13.25 (4)_	Mn_28_Co_32.32 (6)_Ge_13.68 (6)_
Empirical formula	Mn_14_Co_12_Ge_5_(Mn_0.04_Co_0.15_Ge_0.07_)_23_	Mn_14_Co_14_Ge_5_(Co_0.096_Ge_0.08_)_23_
Sample code	10	7
Calculated composition	Mn_40.2_Co_41.9_Ge_17.9_	Mn_37.8_Co_43.9_Ge_18.3_
EDS composition	Mn_40.4 (5)_Co_42.0 (7)_Ge_17.6 (3)_	Mn_37.7 (9)_Co_45.1 (9)_Ge_17.2 (5)_
CSD	2057512	2057511
Structure type relation	Mn_14_(Mn_0.11_Co_0.64_Si_0.25_)_23_	Mn_14_(Mn_0.11_Co_0.64_Si_0.25_)_23_
Formula weight, *M*_r_ (g mol^−1^)	4422.90	4436.02
Space group (No.)	*Pnnm* (58)	*Pnnm* (58)
Pearson symbol, *Z*	*oP*74, 1	*oP*74, 1
Unit-cell dimensions:		
*a* (Å)	12.6427 (10)	12.6208 (12)
*b* (Å)	15.6725 (12)	15.6878 (15)
*c* (Å)	4.8374 (4)	4.8338 (5)
*V* (Å^3^)	958.50 (13)	957.06 (16)
Calculated density, ρ (g cm^−3^)	7.66	7.70
Absorption coefficient, μ (mm^−1^)	32.54	32.93
Theta range for data collection (°)	2.070–42.410	2.071–46.877
*F*(000)	2005	2010
Range in *h k l*	−23 ≤ *h* ≤ 23	−24 ≤ *h* ≤ 25
	−29 ≤ *k* ≤ 29	−25 ≤ *k* ≤ 32
	−9 ≤ *l* ≤ 9	−9 ≤ *l* ≤ 9
Total No. of reflections	22326	38733
*R*_int_/*R*_σ_	0.0299/0.0217	0.0411/0.0255
No. of independent reflections	3686	4648
No. of reflections with *I* > 2σ(*I*)	3277	4313
Data/parameters	3686/106	4648/106
Goodness-of-fit on *F* ^2^	1.103	1.219
Final *R* indices [*I* > 2σ(*I*)]	*R*_1_ = 0.0219	*R*_1_ = 0.0323
	w*R*_2_ = 0.0502	w*R*_2_ = 0.0779
*R* indices (all data)	*R*_1_ = 0.0265	*R*_1_ = 0.0359
	w*R*_2_ = 0.0515	w*R*_2_ = 0.0795
Largest diff. peak and hole (e Å^−3^)	1.241 and −0.919	2.257 and −1.675

Computer programs: *APEX3* (Bruker, 2015 ▸), *SAINT* (Bruker, 2015 ▸), *SHELXT2014* (Sheldrick, 2015*a* ▸), *SHELXL2018* (Sheldrick, 2015*b* ▸), *DIAMOND* (Brandenburg and Putz, 2006 ▸) and *FULLPROF* (Rodriguez-Carvajal, 2001 ▸).

**Table 2:** Atomic coordinates and equivalent isotropic displacement parameters for the Mn_14.9_Co_15.5_Ge_6.6_ and Mn_14_Co_16.2_Ge_6.8_ com­pounds *U*_eq_ is defined as one-third of the trace of the orthogonalized *U_ij_* tensor. For Mn_14.89 (5)_Co_15.48 (4)_Ge_6.62 (2)_, *M*1 and *M*3 are 0.876 (12)Co + 0.124 (12)Mn and 0.90Co + 0.10Mn, respectively, and *M*2 = 0.594 (5)Co + 0.406 (5)Ge. For Mn_14_Co_16.16 (3)_Ge_6.84 (3)_, *M*2 = 0.540 (7)Co + 0.460 (7)Ge.

Mn_14.89 (5)_Co_15.48 (4)_Ge_6.62 (2)_						Mn_14_Co_16.16 (3)_Ge_6.84 (3)_					
Atom	Site	*x*	*y*	*z*	*U*_eq_ (Å^2^)	Atom	Site	*x*	*y*	*z*	*U*_eq_ (Å^2^)
Mn1	4*g*	0.02666 (3)	0.57115 (2)	0	0.00762 (5)	Mn1	4*g*	0.02630 (3)	0.57128 (2)	0	0.00617 (6)
Mn2	4*g*	0.08586 (3)	0.73416 (2)	0	0.00681 (5)	Mn2	4*g*	0.08564 (3)	0.73405 (2)	0	0.00553 (5)
Mn3	4*g*	0.10176 (3)	0.16147 (2)	0	0.00729 (5)	Mn3	4*g*	0.10193 (3)	0.16150 (3)	0	0.00594 (6)
Mn4	4*g*	0.22070 (3)	0.44440 (2)	0	0.00763 (5)	Mn4	4*g*	0.22075 (3)	0.44428 (2)	0	0.00622 (6)
Mn5	4*g*	0.28518 (3)	0.26433 (2)	0	0.00710 (5)	Mn5	4*g*	0.28525 (3)	0.26426 (2)	0	0.00566 (6)
Mn6	4*g*	0.40147 (3)	0.54997 (2)	0	0.00673 (5)	Mn6	4*g*	0.40142 (3)	0.54991 (3)	0	0.00543 (6)
Mn7	4*g*	0.59950 (3)	0.02711 (2)	0	0.00717 (5)	Mn7	4*g*	0.59952 (3)	0.02717 (2)	0	0.00573 (6)
*M*1	8*h*	0.10076 (2)	0.32411 (2)	0.23460 (4)	0.00519 (4)	Co1	8*h*	0.10096 (2)	0.32409 (2)	0.23463 (5)	0.00405 (4)
*M*2	8*h*	0.28921 (2)	0.10244 (2)	0.25373 (4)	0.00601 (5)	*M*2	8*h*	0.28931 (2)	0.10233 (2)	0.25377 (4)	0.00490 (5)
*M*3	8*h*	0.41242 (2)	0.39078 (2)	0.24024 (4)	0.00525 (4)	Co3	8*h*	0.41258 (2)	0.39066 (2)	0.24029 (4)	0.00417 (4)
Co4	4*g*	0.19010 (2)	0.00139 (2)	0	0.00605 (5)	Co4	4*g*	0.19001 (3)	0.00128 (2)	0	0.00458 (5)
Co5	4*g*	0.44439 (3)	0.13693 (2)	0	0.00660 (5)	Co5	4*g*	0.44448 (3)	0.13683 (2)	0	0.00501 (5)
Co6	4*g*	0.69785 (3)	0.29091 (2)	0	0.00626 (5)	Co6	4*g*	0.69799 (3)	0.29094 (2)	0	0.00482 (5)
Ge1	4*g*	0.50766 (2)	0.28512 (2)	0	0.00626 (4)	Ge1	4*g*	0.50782 (2)	0.28499 (2)	0	0.00489 (4)
Ge2	4*g*	0.75789 (2)	0.14623 (2)	0	0.00624 (4)	Ge2	4*g*	0.75801 (2)	0.14630 (2)	0	0.00472 (5)
Ge3	2*a*	0	0	0	0.00616 (5)	Ge3	2*a*	0	0	0	0.00474 (6)
