# Supplementary material for: Variants of the X-phase in the Mn–Co–Ge system
Source: Acta Crystallogr C Struct Chem. 2021 Mar 9;77(Pt 4):176–80. doi: 10.1107/S2053229621002370 (PMC8020885; doi:10.1107/S2053229621002370)
Supplement: Supplementary file 4 [file c-77-00176-sup4.pdf]

STRUCTURAL CHEMISTRY

Volume 77 (2021)

Supporting information for article:

Variants of the *X*-phase in the Mn–Co–Ge system

Vitalii Shtender, Simon R. Larsen and Martin Sahlberg

Anisotropic displacement parameters for $Mn_{14.9}Co_{15.5}Ge_{6.6}$, in Å^2						
**Atom**	U_{11}	U_{22}	U_{33}	U_{12}	U_{13}	U_{23}
Mn1	0.00776(12)	0.00632(11)	0.00877(12)	-0.0009(1)	0.00000	0.00000
Mn2	0.00568(11)	0.00643(11)	0.00831(12)	-0.00049(9)	0.00000	0.00000
Mn3	0.00671(12)	0.00690(12)	0.00826(11)	0.00025(9)	0.00000	0.00000
Mn4	0.00888(13)	0.00681(11)	0.00721(12)	-0.00104(10)	0.00000	0.00000
Mn5	0.00615(12)	0.00746(11)	0.00771(12)	0.00006(9)	0.00000	0.00000
Mn6	0.00600(11)	0.00692(12)	0.00728(11)	0.00017(9)	0.00000	0.00000
Mn7	0.00517(11)	0.00606(12)	0.01029(12)	-0.00008(9)	0.00000	0.00000
*M*1	0.00467(8)	0.00666(8)	0.00424(7)	-0.00006(6)	0.00005(6)	0.00053(5)
*M*2	0.00573(8)	0.00671(8)	0.00560(7)	-0.00075(5)	0.00089(5)	-0.00113(5)
*M*3	0.00542(7)	0.00591(8)	0.00442(7)	0.00017(6)	0.00076(5)	-0.00078(5)
Co4	0.0045(1)	0.00521(10)	0.00844(11)	-0.00084(8)	0.00000	0.00000
Co5	0.00448(10)	0.00723(11)	0.00807(11)	-0.00127(8)	0.00000	0.00000
Co6	0.00516(10)	0.0054(1)	0.00824(11)	-0.00066(8)	0.00000	0.00000

*M*1 and *M*3 equal to 0.88Co1+0.12Mn11 and 0.9Co3+0.1Mn33 respectively; *M*2 = 0.59Co2+0.41Ge22

Selected geometric informations for $Mn_{14.9}Co_{15.5}Ge_{6.6}$			
**Atoms 1,2**	**d 1,2 [Å]**	**Atoms 1,2**	**d 1,2 [Å]**
Mn1—Mn1^{i}	2.3298(7)	Mn5—M3^{ix}	2.8046(4)
Mn1—M1^{ii}	2.5646(4)	Mn5—M3	2.8046(4)
Mn1—M1^{i}	2.5646(4)	Mn5—Ge2^{vii}	2.8167(3)
Mn1—Co5^{iii}	2.6546(3)	Mn5—Ge2^{vi}	2.8167(3)
Mn1—Co5^{iv}	2.6546(3)	Mn5—M2^{ix}	2.8190(4)
Mn1—M2^{v}	2.6607(4)	Mn5—M2	2.8190(4)
Mn1—M2^{iv}	2.6607(4)	Mn6—M3^{ix}	2.7556(4)
Mn1—Mn2	2.6621(5)	Mn6—M3	2.7556(4)
Mn1—Mn7^{iv}	2.9783(3)	Mn6—M3^{x}	2.7837(4)
Mn1—Mn7^{iii}	2.9783(3)	Mn6—M3^{viii}	2.7837(4)
Mn1—Mn7^{vi}	3.0117(3)	Mn6—Co4^{iv}	2.7875(3)
Mn1—Mn7^{vii}	3.0117(3)	Mn6—Co4^{iii}	2.7875(3)
Mn2—Ge2^{viii}	2.7233(4)	Mn6—Co6^{viii}	2.7922(5)

Mn2—M3^{v}	2.7577(4)	Mn6—M2^{iv}	2.8120(4)
Mn2—M3^{iv}	2.7577(4)	Mn6—M2^{v}	2.8120(4)
Mn2—Co6^{viii}	2.7626(5)	Mn6—Ge1^{viii}	2.8284(5)
Mn2—M1^{i}	2.7728(4)	Mn7—Co5	2.6091(5)
Mn2—M1^{ii}	2.7728(4)	Mn7—Co5^{xi}	2.6301(5)
Mn2—Ge1^{iv}	2.8082(3)	Mn7—Mn7^{xi}	2.6556(7)
Mn2—Ge1^{iii}	2.8082(3)	Mn7—M1^{xii}	2.6618(4)
Mn2—M2^{iv}	2.8593(4)	Mn7—M1^{xiii}	2.6618(4)
Mn2—M2^{v}	2.8593(4)	Mn7—Co4^{xi}	2.6973(5)
Mn2—Mn3^{i}	2.8813(5)	Mn7—Ge2	2.7377(4)
Mn3—Co4	2.7461(5)	Mn7—M2^{xi}	2.7584(4)
Mn3—M1	2.7903(4)	Mn7—M2^{xiv}	2.7584(4)
Mn3—M1^{ix}	2.7903(4)	M1—M1^{ix}	2.2697(4)
Mn3—Co6^{vii}	2.8077(3)	M1—Ge2^{vii}	2.4105(3)
Mn3—Co6^{vi}	2.8077(3)	M1—Co5^{vii}	2.4350(3)
Mn3—Ge1^{vi}	2.8224(3)	M1—Ge1^{vii}	2.4422(3)
Mn3—Ge1^{vii}	2.8224(3)	M1—Co6^{vii}	2.5307(3)
Mn3—Mn5	2.8243(5)	M1—M1^{xv}	2.5677(4)
Mn3—M2^{ix}	2.8247(4)	M2—Co6^{vii}	2.3552(3)
Mn3—M2	2.8247(4)	M2—Co4	2.3632(3)
Mn4—M1^{ix}	2.6723(4)	M2—Co5	2.3765(3)
Mn4—M1	2.6723(4)	M2—M2^{xv}	2.3826(4)
Mn4—M2^{v}	2.7514(4)	M2—M2^{ix}	2.4548(4)
Mn4—M2^{iv}	2.7514(4)	M3—M3^{ix}	2.3242(4)
Mn4—Co4^{iii}	2.8142(3)	M3—Ge1	2.3543(3)
Mn4—Co4^{iv}	2.8142(3)	M3—Ge2^{vii}	2.3942(3)
Mn4—M3^{ix}	2.8162(4)	M3—Ge3^{xii}	2.3948(2)
Mn4—M3	2.8163(4)	M3—Co4^{iii}	2.5028(3)
Mn4—Mn6	2.8215(5)	M3—M3^{xv}	2.5132(4)
Mn4—Ge2^{vi}	2.8441(3)	Co4—Ge3	2.4035(4)
Mn5—M1^{ix}	2.7571(4)	Co4—Ge2^{xi}	2.4053(4)
Mn5—M1	2.7571(4)	Co5—Ge1	2.4564(4)
Mn5—Co6^{vi}	2.7962(3)	Co6—Ge2	2.3911(4)
Mn5—Co6^{vii}	2.7962(3)	Co6—Ge1	2.4063(4)

Atoms 1,2,3	Angle 1,2,3 [°]	Atoms 1,2,3	Angle 1,2,3 [°]
$Mn1^{i}$—Mn1—$M1^{ii}$	115.53(2)	$Co6^{vii}$—M2—$Mn7^{xi}$	176.035(10)
$Mn1^{i}$—Mn1—$M1^{i}$	115.53(2)	Co4—M2—$Mn7^{xi}$	63.003(11)
$M1^{ii}$—Mn1—$M1^{i}$	52.528(12)	Co5—M2—$Mn7^{xi}$	61.087(11)
$Mn1^{i}$—Mn1—$Co5^{iii}$	114.306(10)	$M2^{xv}$—M2—$Mn7^{xi}$	116.422(6)
$M1^{ii}$—Mn1—$Co5^{iii}$	55.589(9)	$M2^{ix}$—M2—$Mn7^{xi}$	63.579(5)
$M1^{i}$—Mn1—$Co5^{iii}$	103.961(14)	$Mn1^{xviii}$—M2—$Mn7^{xi}$	67.497(10)
$Mn1^{i}$—Mn1—$Co5^{iv}$	114.306(10)	$Mn4^{xviii}$—M2—$Mn7^{xi}$	63.463(9)
$M1^{ii}$—Mn1—$Co5^{iv}$	103.961(14)	$Co6^{vii}$—M2—$Mn6^{xviii}$	64.713(11)
$M1^{i}$—Mn1—$Co5^{iv}$	55.589(10)	Co4—M2—$Mn6^{xviii}$	64.499(9)
$Co5^{iii}$—Mn1—$Co5^{iv}$	131.329(19)	Co5—M2—$Mn6^{xviii}$	173.252(10)
$Mn1^{i}$—Mn1—$M2^{v}$	115.43(2)	$M2^{xv}$—M2—$Mn6^{xviii}$	64.934(5)
$M1^{ii}$—Mn1—$M2^{v}$	103.503(11)	$M2^{ix}$—M2—$Mn6^{xviii}$	115.065(5)
$M1^{i}$—Mn1—$M2^{v}$	129.031(15)	$Mn1^{xviii}$—M2—$Mn6^{xviii}$	120.433(11)
$Co5^{iii}$—Mn1—$M2^{v}$	53.118(9)	$Mn4^{xviii}$—M2—$Mn6^{xviii}$	60.936(11)
$Co5^{iv}$—Mn1—$M2^{v}$	102.459(14)	$Mn7^{xi}$—M2—$Mn6^{xviii}$	114.237(12)
$Mn1^{i}$—Mn1—$M2^{iv}$	115.43(2)	$Co6^{vii}$—M2—Mn5	64.697(9)
$M1^{ii}$—Mn1—$M2^{iv}$	129.031(15)	Co4—M2—Mn5	111.558(11)
$M1^{i}$—Mn1—$M2^{iv}$	103.503(11)	Co5—M2—Mn5	65.503(11)
$Co5^{iii}$—Mn1—$M2^{iv}$	102.459(14)	$M2^{xv}$—M2—Mn5	115.811(5)
$Co5^{iv}$—Mn1—$M2^{iv}$	53.118(9)	$M2^{ix}$—M2—Mn5	64.189(5)
$M2^{v}$—Mn1—$M2^{iv}$	53.197(11)	$Mn1^{xviii}$—M2—Mn5	112.126(12)
$Mn1^{i}$—Mn1—Mn2	179.52(3)	$Mn4^{xviii}$—M2—Mn5	176.351(13)
$M1^{ii}$—Mn1—Mn2	64.048(12)	$Mn7^{xi}$—M2—Mn5	118.556(10)
$M1^{i}$—Mn1—Mn2	64.048(12)	$Mn6^{xviii}$—M2—Mn5	115.607(12)
$Co5^{iii}$—Mn1—Mn2	65.706(10)	$Co6^{vii}$—M2—Mn3	64.907(9)
$Co5^{iv}$—Mn1—Mn2	65.706(10)	Co4—M2—Mn3	63.187(12)
$M2^{v}$—Mn1—Mn2	64.983(11)	Co5—M2—Mn3	113.187(11)
$M2^{iv}$—Mn1—Mn2	64.983(11)	$M2^{xv}$—M2—Mn3	115.754(5)
$Mn1^{i}$—Mn1—$Mn7^{iv}$	67.870(11)	$M2^{ix}$—M2—Mn3	64.244(6)

$M1^{ii}$—Mn1—$Mn7^{iv}$	99.865(14)	$Mn1^{xviii}$—M2—Mn3	171.499(13)
$M1^{i}$—Mn1—$Mn7^{iv}$	56.811(10)	$Mn4^{xviii}$—M2—Mn3	116.408(12)
$Co5^{iii}$—Mn1—$Mn7^{iv}$	154.660(16)	$Mn7^{xi}$—M2—Mn3	118.403(10)
$Co5^{iv}$—Mn1—$Mn7^{iv}$	54.822(10)	$Mn6^{xviii}$—M2—Mn3	63.933(10)
$M2^{v}$—Mn1—$Mn7^{iv}$	151.030(11)	Mn5—M2—Mn3	60.058(10)
$M2^{iv}$—Mn1—$Mn7^{iv}$	98.491(9)	$Co6^{vii}$—M2—$Mn2^{xviii}$	63.112(12)
Mn2—Mn1—$Mn7^{iv}$	111.899(11)	Co4—M2—$Mn2^{xviii}$	173.281(9)
$Mn1^{i}$—Mn1—$Mn7^{iii}$	67.870(11)	Co5—M2—$Mn2^{xviii}$	66.106(9)
$M1^{ii}$—Mn1—$Mn7^{iii}$	56.811(10)	$M2^{xv}$—M2—$Mn2^{xviii}$	65.378(5)
$M1^{i}$—Mn1—$Mn7^{iii}$	99.865(14)	$M2^{ix}$—M2—$Mn2^{xviii}$	114.623(5)
$Co5^{iii}$—Mn1—$Mn7^{iii}$	54.822(10)	$Mn1^{xviii}$—M2—$Mn2^{xviii}$	57.534(11)
$Co5^{iv}$—Mn1—$Mn7^{iii}$	154.660(16)	$Mn4^{xviii}$—M2—$Mn2^{xviii}$	119.653(11)
$M2^{v}$—Mn1—$Mn7^{iii}$	98.491(9)	$Mn7^{xi}$—M2—$Mn2^{xviii}$	115.791(13)
$M2^{iv}$—Mn1—$Mn7^{iii}$	151.030(11)	$Mn6^{xviii}$—M2—$Mn2^{xviii}$	120.545(10)
Mn2—Mn1—$Mn7^{iii}$	111.899(11)	Mn5—M2—$Mn2^{xviii}$	62.713(9)
$Mn7^{iv}$—Mn1—$Mn7^{iii}$	108.605(16)	Mn3—M2—$Mn2^{xviii}$	114.082(12)
$Mn1^{i}$—Mn1—$Mn7^{vi}$	66.355(11)	$M3^{ix}$—M3—Ge1	60.422(6)
$M1^{ii}$—Mn1—$Mn7^{vi}$	151.018(12)	$M3^{ix}$—M3—$Ge2^{vii}$	121.656(6)
$M1^{i}$—Mn1—$Mn7^{vi}$	99.440(9)	Ge1—M3—$Ge2^{vii}$	120.393(12)
$Co5^{iii}$—Mn1—$Mn7^{vi}$	152.641(16)	$M3^{ix}$—M3—$Ge3^{xii}$	121.650(6)
$Co5^{iv}$—Mn1—$Mn7^{vi}$	54.876(10)	Ge1—M3—$Ge3^{xii}$	121.689(12)
$M2^{v}$—Mn1—$Mn7^{vi}$	100.733(13)	$Ge2^{vii}$—M3—$Ge3^{xii}$	105.968(10)
$M2^{iv}$—Mn1—$Mn7^{vi}$	57.797(9)	$M3^{ix}$—M3—$Co4^{iii}$	120.136(6)
Mn2—Mn1—$Mn7^{vi}$	113.875(11)	Ge1—M3—$Co4^{iii}$	179.133(12)
$Mn7^{iv}$—Mn1—$Mn7^{vi}$	52.632(12)	$Ge2^{vii}$—M3—$Co4^{iii}$	58.786(10)
$Mn7^{iii}$—Mn1—$Mn7^{vi}$	134.225(13)	$Ge3^{xii}$—M3—$Co4^{iii}$	58.730(9)
$Mn1^{i}$—Mn1—$Mn7^{vii}$	66.355(12)	$M3^{ix}$—M3—$M3^{xv}$	180.000
$M1^{ii}$—Mn1—$Mn7^{vii}$	99.440(9)	Ge1—M3—$M3^{xv}$	119.578(6)
$M1^{i}$—Mn1—$Mn7^{vii}$	151.018(12)	$Ge2^{vii}$—M3—$M3^{xv}$	58.342(6)
$Co5^{iii}$—Mn1—$Mn7^{vii}$	54.876(10)	$Ge3^{xii}$—M3—$M3^{xv}$	58.351(5)
$Co5^{iv}$—Mn1—$Mn7^{vii}$	152.641(16)	$Co4^{iii}$—M3—$M3^{xv}$	59.863(6)
$M2^{v}$—Mn1—$Mn7^{vii}$	57.797(9)	$M3^{ix}$—M3—Mn6	65.056(6)
$M2^{iv}$—Mn1—$Mn7^{vii}$	100.733(13)	Ge1—M3—Mn6	117.022(11)
Mn2—Mn1—$Mn7^{vii}$	113.875(11)	$Ge2^{vii}$—M3—Mn6	113.562(12)

$Mn7^{iv}$—Mn1—$Mn7^{vii}$	134.225(13)	$Ge3^{xii}$—M3—Mn6	66.260(8)
$Mn7^{iii}$—Mn1—$Mn7^{vii}$	52.632(12)	$Co4^{iii}$—M3—Mn6	63.811(9)
$Mn7^{vi}$—Mn1—$Mn7^{vii}$	106.855(16)	$M3^{xv}$—M3—Mn6	114.944(6)
Mn1—Mn2—$Ge2^{viii}$	149.830(17)	$M3^{ix}$—M3—$Mn2^{xviii}$	117.108(6)
Mn1—Mn2—$M3^{v}$	148.918(10)	Ge1—M3—$Mn2^{xviii}$	66.094(9)
$Ge2^{viii}$—Mn2—$M3^{v}$	51.797(9)	$Ge2^{vii}$—M3—$Mn2^{xviii}$	63.359(10)
Mn1—Mn2—$M3^{iv}$	148.918(10)	$Ge3^{xii}$—M3—$Mn2^{xviii}$	113.171(10)
$Ge2^{viii}$—Mn2—$M3^{iv}$	51.797(9)	$Co4^{iii}$—M3—$Mn2^{xviii}$	113.070(11)
$M3^{v}$—Mn2—$M3^{iv}$	54.215(12)	$M3^{xv}$—M3—$Mn2^{xviii}$	62.893(6)
Mn1—Mn2—$Co6^{viii}$	98.155(15)	Mn6—M3—$Mn2^{xviii}$	176.772(11)
$Ge2^{viii}$—Mn2—$Co6^{viii}$	51.675(11)	$M3^{ix}$—M3—$Mn6^{viii}$	65.326(6)
$M3^{v}$—Mn2—$Co6^{viii}$	96.820(12)	Ge1—M3—$Mn6^{viii}$	66.190(11)
$M3^{iv}$—Mn2—$Co6^{viii}$	96.820(12)	$Ge2^{vii}$—M3—$Mn6^{viii}$	171.747(11)
Mn1—Mn2—$M1^{i}$	56.266(10)	$Ge3^{xii}$—M3—$Mn6^{viii}$	65.783(8)
$Ge2^{viii}$—Mn2—$M1^{i}$	147.559(11)	$Co4^{iii}$—M3—$Mn6^{viii}$	114.599(13)
$M3^{v}$—Mn2—$M1^{i}$	119.104(14)	$M3^{xv}$—M3—$Mn6^{viii}$	114.676(5)
$M3^{iv}$—Mn2—$M1^{i}$	96.513(10)	Mn6—M3—$Mn6^{viii}$	64.175(13)
$Co6^{viii}$—Mn2—$M1^{i}$	142.693(13)	$Mn2^{xviii}$—M3—$Mn6^{viii}$	118.717(12)
Mn1—Mn2—$M1^{ii}$	56.266(10)	$M3^{ix}$—M3—Mn5	65.520(6)
$Ge2^{viii}$—Mn2—$M1^{ii}$	147.559(11)	Ge1—M3—Mn5	65.905(11)
$M3^{v}$—Mn2—$M1^{ii}$	96.513(10)	$Ge2^{vii}$—M3—Mn5	65.053(9)
$M3^{iv}$—Mn2—$M1^{ii}$	119.104(14)	$Ge3^{xii}$—M3—Mn5	171.003(10)
$Co6^{viii}$—Mn2—$M1^{ii}$	142.693(12)	$Co4^{iii}$—M3—Mn5	113.606(13)
$M1^{i}$—Mn2—$M1^{ii}$	48.318(11)	$M3^{xv}$—M3—Mn5	114.479(5)
Mn1—Mn2—$Ge1^{iv}$	98.89(1)	Mn6—M3—Mn5	115.861(11)
$Ge2^{viii}$—Mn2—$Ge1^{iv}$	96.295(10)	$Mn2^{xviii}$—M3—Mn5	64.174(10)
$M3^{v}$—Mn2—$Ge1^{iv}$	98.201(12)	$Mn6^{viii}$—M3—Mn5	123.198(10)
$M3^{iv}$—Mn2—$Ge1^{iv}$	50.038(7)	$M3^{ix}$—M3—Mn4	65.628(6)
$Co6^{viii}$—Mn2—$Ge1^{iv}$	117.206(9)	Ge1—M3—Mn4	116.509(10)
$M1^{i}$—Mn2—$Ge1^{iv}$	51.896(8)	$Ge2^{vii}$—M3—Mn4	65.581(9)
$M1^{ii}$—Mn2—$Ge1^{iv}$	95.045(13)	$Ge3^{xii}$—M3—Mn4	113.654(10)
Mn1—Mn2—$Ge1^{iii}$	98.89(1)	$Co4^{iii}$—M3—Mn4	63.566(9)
$Ge2^{viii}$—Mn2—$Ge1^{iii}$	96.295(10)	$M3^{xv}$—M3—Mn4	114.370(6)
$M3^{v}$—Mn2—$Ge1^{iii}$	50.038(8)	Mn6—M3—Mn4	60.834(11)

M3^{iv}—Mn2—Ge1^{iii}	98.201(12)	Mn2^{xviii}—M3—Mn4	117.412(12)
Co6^{viii}—Mn2—Ge1^{iii}	117.206(8)	Mn6^{viii}—M3—Mn4	117.108(11)
M1^{i}—Mn2—Ge1^{iii}	95.045(12)	Mn5—M3—Mn4	63.012(11)
M1^{ii}—Mn2—Ge1^{iii}	51.896(8)	M3^{ix}—M3—Mn3^{xii}	116.414(6)
Ge1^{iv}—Mn2—Ge1^{iii}	118.928(16)	Ge1—M3—Mn3^{xii}	65.309(9)
Mn1—Mn2—M2^{iv}	57.485(11)	Ge2^{vii}—M3—Mn3^{xii}	112.814(11)
Ge2^{viii}—Mn2—M2^{iv}	95.523(13)	Ge3^{xii}—M3—Mn3^{xii}	65.291(9)
M3^{v}—Mn2—M2^{iv}	145.906(15)	Co4^{iii}—M3—Mn3^{xii}	114.601(11)
M3^{iv}—Mn2—M2^{iv}	116.643(10)	M3^{xv}—M3—Mn3^{xii}	63.588(6)
Co6^{viii}—Mn2—M2^{iv}	49.498(9)	Mn6—M3—Mn3^{xii}	119.512(12)
M1^{i}—Mn2—M2^{iv}	93.539(11)	Mn2^{xviii}—M3—Mn3^{xii}	62.132(11)
M1^{ii}—Mn2—M2^{iv}	113.751(14)	Mn6^{viii}—M3—Mn3^{xii}	64.289(10)
Ge1^{iv}—Mn2—M2^{iv}	94.534(8)	Mn5—M3—Mn3^{xii}	117.743(12)
Ge1^{iii}—Mn2—M2^{iv}	142.822(11)	Mn4—M3—Mn3^{xii}	177.948(9)
Mn1—Mn2—M2^{v}	57.485(11)	M2^{ix}—Co4—M2	62.582(12)
Ge2^{viii}—Mn2—M2^{v}	95.523(13)	M2^{ix}—Co4—Ge3	122.431(12)
M3^{v}—Mn2—M2^{v}	116.643(10)	M2—Co4—Ge3	122.432(12)
M3^{iv}—Mn2—M2^{v}	145.906(15)	M2^{ix}—Co4—Ge2^{xi}	119.978(13)
Co6^{viii}—Mn2—M2^{v}	49.498(9)	M2—Co4—Ge2^{xi}	119.977(13)
M1^{i}—Mn2—M2^{v}	113.751(14)	Ge3—Co4—Ge2^{xi}	105.345(12)
M1^{ii}—Mn2—M2^{v}	93.539(11)	M2^{ix}—Co4—M3^{xvii}	118.553(8)
Ge1^{iv}—Mn2—M2^{v}	142.822(11)	M2—Co4—M3^{xvii}	178.226(13)
Ge1^{iii}—Mn2—M2^{v}	94.534(8)	Ge3—Co4—M3^{xvii}	58.391(8)
M2^{iv}—Mn2—M2^{v}	49.245(10)	Ge2^{xi}—Co4—M3^{xvii}	58.353(10)
Mn1—Mn2—Mn3^{i}	108.261(16)	M2^{ix}—Co4—M3^{xix}	178.226(13)
Ge2^{viii}—Mn2—Mn3^{i}	101.909(15)	M2—Co4—M3^{xix}	118.553(8)
M3^{v}—Mn2—Mn3^{i}	60.078(10)	Ge3—Co4—M3^{xix}	58.391(8)
M3^{iv}—Mn2—Mn3^{i}	60.078(10)	Ge2^{xi}—Co4—M3^{xix}	58.353(10)
Co6^{viii}—Mn2—Mn3^{i}	153.584(17)	M3^{xvii}—Co4—M3^{xix}	60.276(12)
M1^{i}—Mn2—Mn3^{i}	59.104(11)	M2^{ix}—Co4—Mn7^{xi}	65.676(11)
M1^{ii}—Mn2—Mn3^{i}	59.104(11)	M2—Co4—Mn7^{xi}	65.676(11)
Ge1^{iv}—Mn2—Mn3^{i}	59.466(8)	Ge3—Co4—Mn7^{xi}	169.945(15)
Ge1^{iii}—Mn2—Mn3^{i}	59.466(8)	Ge2^{xi}—Co4—Mn7^{xi}	64.599(12)
M2^{iv}—Mn2—Mn3^{i}	149.842(10)	M3^{xvii}—Co4—Mn7^{xi}	113.326(13)

M2^{v}—Mn2—Mn3^{i}	149.842(10)	M3^{xix}—Co4—Mn7^{xi}	113.326(13)
Co4—Mn3—M1	146.763(12)	M2^{ix}—Co4—Mn3	66.636(11)
Co4—Mn3—M1^{ix}	146.763(12)	M2—Co4—Mn3	66.637(11)
M1—Mn3—M1^{ix}	47.997(11)	Ge3—Co4—Mn3	66.522(10)
Co4—Mn3—Co6^{vii}	93.834(11)	Ge2^{xi}—Co4—Mn3	171.867(16)
M1—Mn3—Co6^{vii}	53.753(9)	M3^{xvii}—Co4—Mn3	114.970(13)
M1^{ix}—Mn3—Co6^{vii}	96.284(13)	M3^{xix}—Co4—Mn3	114.970(13)
Co4—Mn3—Co6^{vi}	93.834(11)	Mn7^{xi}—Co4—Mn3	123.533(15)
M1—Mn3—Co6^{vi}	96.284(13)	M2^{ix}—Co4—Mn6^{xvii}	65.577(10)
M1^{ix}—Mn3—Co6^{vi}	53.753(9)	M2—Co4—Mn6^{xvii}	119.202(14)
Co6^{vii}—Mn3—Co6^{vi}	118.959(17)	Ge3—Co4—Mn6^{xvii}	65.617(9)
Co4—Mn3—Ge1^{vi}	116.257(9)	Ge2^{xi}—Co4—Mn6^{xvii}	112.101(10)
M1—Mn3—Ge1^{vi}	94.339(12)	M3^{xvii}—Co4—Mn6^{xvii}	62.511(10)
M1^{ix}—Mn3—Ge1^{vi}	51.582(8)	M3^{xix}—Co4—Mn6^{xvii}	114.188(14)
Co6^{vii}—Mn3—Ge1^{vi}	147.326(16)	Mn7^{xi}—Co4—Mn6^{xvii}	117.054(9)
Co6^{vi}—Mn3—Ge1^{vi}	50.603(9)	Mn3—Co4—Mn6^{xvii}	65.266(9)
Co4—Mn3—Ge1^{vii}	116.257(8)	M2^{ix}—Co4—Mn6^{xviii}	119.201(14)
M1—Mn3—Ge1^{vii}	51.581(8)	M2—Co4—Mn6^{xviii}	65.577(10)
M1^{ix}—Mn3—Ge1^{vii}	94.339(12)	Ge3—Co4—Mn6^{xviii}	65.617(9)
Co6^{vii}—Mn3—Ge1^{vii}	50.603(9)	Ge2^{xi}—Co4—Mn6^{xviii}	112.101(10)
Co6^{vi}—Mn3—Ge1^{vii}	147.326(16)	M3^{xvii}—Co4—Mn6^{xviii}	114.188(13)
Ge1^{vi}—Mn3—Ge1^{vii}	117.952(15)	M3^{xix}—Co4—Mn6^{xviii}	62.511(10)
Co4—Mn3—Mn5	100.809(15)	Mn7^{xi}—Co4—Mn6^{xviii}	117.054(9)
M1—Mn3—Mn5	58.817(10)	Mn3—Co4—Mn6^{xviii}	65.266(9)
M1^{ix}—Mn3—Mn5	58.817(10)	Mn6^{xvii}—Co4—Mn6^{xviii}	120.387(17)
Co6^{vii}—Mn3—Mn5	59.534(9)	M2^{ix}—Co4—Mn4^{xviii}	116.529(14)
Co6^{vi}—Mn3—Mn5	59.534(8)	M2—Co4—Mn4^{xviii}	63.503(10)
Ge1^{vi}—Mn3—Mn5	100.181(10)	Ge3—Co4—Mn4^{xviii}	113.442(9)
Ge1^{vii}—Mn3—Mn5	100.181(11)	Ge2^{xi}—Co4—Mn4^{xviii}	65.489(9)
Co4—Mn3—M2^{ix}	50.176(9)	M3^{xvii}—Co4—Mn4^{xviii}	114.783(13)
M1—Mn3—M2^{ix}	118.666(13)	M3^{xix}—Co4—Mn4^{xviii}	63.649(10)
M1^{ix}—Mn3—M2^{ix}	97.253(10)	Mn7^{xi}—Co4—Mn4^{xviii}	63.400(9)
Co6^{vii}—Mn3—M2^{ix}	95.644(13)	Mn3—Co4—Mn4^{xviii}	116.933(9)
Co6^{vi}—Mn3—M2^{ix}	49.435(8)	Mn6^{xvii}—Co4—Mn4^{xviii}	177.285(15)

Ge1^{vi}—Mn3—M2^{ix}	94.497(8)	Mn6^{xviii}—Co4—Mn4^{xviii}	60.485(10)
Ge1^{vii}—Mn3—M2^{ix}	145.401(11)	M2—Co5—M2^{ix}	62.191(13)
Mn5—Mn3—M2^{ix}	59.872(11)	M2—Co5—M1^{xii}	117.062(9)
Co4—Mn3—M2	50.176(9)	M2^{ix}—Co5—M1^{xii}	178.331(15)
M1—Mn3—M2	97.253(10)	M2—Co5—M1^{xiii}	178.332(15)
M1^{ix}—Mn3—M2	118.665(13)	M2^{ix}—Co5—M1^{xiii}	117.063(9)
Co6^{vii}—Mn3—M2	49.435(8)	M1^{xii}—Co5—M1^{xiii}	63.639(13)
Co6^{vi}—Mn3—M2	95.644(13)	M2—Co5—Ge1	118.937(12)
Ge1^{vi}—Mn3—M2	145.400(11)	M2^{ix}—Co5—Ge1	118.937(12)
Ge1^{vii}—Mn3—M2	94.497(8)	M1^{xii}—Co5—Ge1	59.904(9)
Mn5—Mn3—M2	59.872(11)	M1^{xiii}—Co5—Ge1	59.904(9)
M2^{ix}—Mn3—M2	51.510(11)	M2—Co5—Mn7	118.064(13)
M1^{ix}—Mn4—M1	50.260(12)	M2^{ix}—Co5—Mn7	118.064(13)
M1^{ix}—Mn4—M2^{v}	142.479(16)	M1^{xii}—Co5—Mn7	63.591(11)
M1—Mn4—M2^{v}	115.174(11)	M1^{xiii}—Co5—Mn7	63.591(11)
M1^{ix}—Mn4—M2^{iv}	115.174(11)	Ge1—Co5—Mn7	112.269(15)
M1—Mn4—M2^{iv}	142.478(16)	M2—Co5—Mn7^{xi}	66.64(1)
M2^{v}—Mn4—M2^{iv}	51.314(11)	M2^{ix}—Co5—Mn7^{xi}	66.64(1)
M1^{ix}—Mn4—Co4^{iii}	144.716(12)	M1^{xii}—Co5—Mn7^{xi}	114.609(13)
M1—Mn4—Co4^{iii}	94.953(8)	M1^{xiii}—Co5—Mn7^{xi}	114.609(13)
M2^{v}—Mn4—Co4^{iii}	50.234(8)	Ge1—Co5—Mn7^{xi}	173.179(16)
M2^{iv}—Mn4—Co4^{iii}	96.005(13)	Mn7—Co5—Mn7^{xi}	60.909(15)
M1^{ix}—Mn4—Co4^{iv}	94.952(9)	M2—Co5—Mn1^{xvii}	119.743(14)
M1—Mn4—Co4^{iv}	144.716(11)	M2^{ix}—Co5—Mn1^{xvii}	63.575(10)
M2^{v}—Mn4—Co4^{iv}	96.005(13)	M1^{xii}—Co5—Mn1^{xvii}	117.763(14)
M2^{iv}—Mn4—Co4^{iv}	50.234(8)	M1^{xiii}—Co5—Mn1^{xvii}	60.331(10)
Co4^{iii}—Mn4—Co4^{iv}	118.511(17)	Ge1—Co5—Mn1^{xvii}	108.80(1)
M1^{ix}—Mn4—M3^{ix}	95.892(11)	Mn7—Co5—Mn1^{xvii}	68.911(10)
M1—Mn4—M3^{ix}	116.945(14)	Mn7^{xi}—Co5—Mn1^{xvii}	69.483(10)
M2^{v}—Mn4—M3^{ix}	119.107(14)	M2—Co5—Mn1^{xviii}	63.574(10)
M2^{iv}—Mn4—M3^{ix}	97.418(10)	M2^{ix}—Co5—Mn1^{xviii}	119.743(14)
Co4^{iii}—Mn4—M3^{ix}	95.997(13)	M1^{xii}—Co5—Mn1^{xviii}	60.331(10)
Co4^{iv}—Mn4—M3^{ix}	52.784(9)	M1^{xiii}—Co5—Mn1^{xviii}	117.763(14)
M1^{ix}—Mn4—M3	116.945(14)	Ge1—Co5—Mn1^{xviii}	108.80(1)

M1—Mn4—M3	95.893(11)	Mn7—Co5—Mn1^{xviii}	68.911(10)
M2^{v}—Mn4—M3	97.418(10)	Mn7^{xi}—Co5—Mn1^{xviii}	69.483(10)
M2^{iv}—Mn4—M3	119.107(14)	Mn1^{xvii}—Co5—Mn1^{xviii}	131.327(19)
Co4^{iii}—Mn4—M3	52.784(9)	M2—Co5—Mn5	64.791(11)
Co4^{iv}—Mn4—M3	95.997(13)	M2^{ix}—Co5—Mn5	64.791(11)
M3^{ix}—Mn4—M3	48.742(11)	M1^{xii}—Co5—Mn5	113.564(13)
M1^{ix}—Mn4—Mn6	150.846(10)	M1^{xiii}—Co5—Mn5	113.564(13)
M1—Mn4—Mn6	150.847(10)	Ge1—Co5—Mn5	64.232(12)
M2^{v}—Mn4—Mn6	60.592(11)	Mn7—Co5—Mn5	176.502(16)
M2^{iv}—Mn4—Mn6	60.592(10)	Mn7^{xi}—Co5—Mn5	122.589(16)
Co4^{iii}—Mn4—Mn6	59.288(8)	Mn1^{xvii}—Co5—Mn5	111.802(10)
Co4^{iv}—Mn4—Mn6	59.288(8)	Mn1^{xviii}—Co5—Mn5	111.802(10)
M3^{ix}—Mn4—Mn6	58.521(11)	M2—Co5—Mn2^{xviii}	65.012(10)
M3—Mn4—Mn6	58.521(11)	M2^{ix}—Co5—Mn2^{xviii}	116.346(14)
M1^{ix}—Mn4—Ge2^{vi}	51.708(8)	M1^{xii}—Co5—Mn2^{xviii}	62.162(10)
M1—Mn4—Ge2^{vi}	95.891(12)	M1^{xiii}—Co5—Mn2^{xviii}	114.666(13)
M2^{v}—Mn4—Ge2^{vi}	145.625(11)	Ge1—Co5—Mn2^{xviii}	62.846(9)
M2^{iv}—Mn4—Ge2^{vi}	95.101(8)	Mn7—Co5—Mn2^{xviii}	116.628(9)
Co4^{iii}—Mn4—Ge2^{vi}	145.407(16)	Mn7^{xi}—Co5—Mn2^{xviii}	119.241(9)
Co4^{iv}—Mn4—Ge2^{vi}	50.310(8)	Mn1^{xvii}—Co5—Mn2^{xviii}	170.950(14)
M3^{ix}—Mn4—Ge2^{vi}	50.043(8)	Mn1^{xviii}—Co5—Mn2^{xviii}	57.275(10)
M3—Mn4—Ge2^{vi}	93.420(12)	Mn5—Co5—Mn2^{xviii}	62.209(9)
Mn6—Mn4—Ge2^{vi}	99.146(10)	M2^{xii}—Co6—M2^{xiii}	60.771(13)
M1^{ix}—Mn5—M1	48.611(11)	M2^{xii}—Co6—Ge2	121.153(13)
M1^{ix}—Mn5—Co6^{vi}	54.217(9)	M2^{xiii}—Co6—Ge2	121.153(13)
M1—Mn5—Co6^{vi}	97.317(13)	M2^{xii}—Co6—Ge1	121.118(13)
M1^{ix}—Mn5—Co6^{vii}	97.317(13)	M2^{xiii}—Co6—Ge1	121.118(13)
M1—Mn5—Co6^{vii}	54.217(9)	Ge2—Co6—Ge1	106.347(14)
Co6^{vi}—Mn5—Co6^{vii}	119.766(17)	M2^{xii}—Co6—M1^{xiii}	179.649(16)
M1^{ix}—Mn5—M3^{ix}	94.265(11)	M2^{xiii}—Co6—M1^{xiii}	119.129(8)
M1—Mn5—M3^{ix}	114.550(14)	Ge2—Co6—M1^{xiii}	58.568(10)
Co6^{vi}—Mn5—M3^{ix}	94.982(9)	Ge1—Co6—M1^{xiii}	59.232(9)
Co6^{vii}—Mn5—M3^{ix}	143.484(12)	M2^{xii}—Co6—M1^{xii}	119.129(8)
M1^{ix}—Mn5—M3	114.550(14)	M2^{xiii}—Co6—M1^{xii}	179.649(16)

M1—Mn5—M3	94.266(11)	Ge2—Co6—M1^{xii}	58.568(10)
Co6^{vi}—Mn5—M3	143.484(11)	Ge1—Co6—M1^{xii}	59.232(9)
Co6^{vii}—Mn5—M3	94.982(9)	M1^{xiii}—Co6—M1^{xii}	60.970(12)
M3^{ix}—Mn5—M3	48.958(11)	M2^{xii}—Co6—Mn2^{viii}	67.390(11)
M1^{ix}—Mn5—Ge2^{vii}	94.633(12)	M2^{xiii}—Co6—Mn2^{viii}	67.390(11)
M1—Mn5—Ge2^{vii}	51.236(8)	Ge2—Co6—Mn2^{viii}	63.315(11)
Co6^{vi}—Mn5—Ge2^{vii}	148.049(16)	Ge1—Co6—Mn2^{viii}	169.662(16)
Co6^{vii}—Mn5—Ge2^{vii}	50.427(9)	M1^{xiii}—Co6—Mn2^{viii}	112.260(13)
M3^{ix}—Mn5—Ge2^{vii}	94.269(12)	M1^{xii}—Co6—Mn2^{viii}	112.260(13)
M3—Mn5—Ge2^{vii}	50.417(8)	M2^{xii}—Co6—Mn6^{viii}	65.585(11)
M1^{ix}—Mn5—Ge2^{vi}	51.235(8)	M2^{xiii}—Co6—Mn6^{viii}	65.585(11)
M1—Mn5—Ge2^{vi}	94.633(12)	Ge2—Co6—Mn6^{viii}	171.782(16)
Co6^{vi}—Mn5—Ge2^{vi}	50.427(9)	Ge1—Co6—Mn6^{viii}	65.435(12)
Co6^{vii}—Mn5—Ge2^{vi}	148.049(16)	M1^{xiii}—Co6—Mn6^{viii}	114.712(13)
M3^{ix}—Mn5—Ge2^{vi}	50.417(8)	M1^{xii}—Co6—Mn6^{viii}	114.712(13)
M3—Mn5—Ge2^{vi}	94.269(12)	Mn2^{viii}—Co6—Mn6^{viii}	124.903(15)
Ge2^{vii}—Mn5—Ge2^{vi}	118.343(15)	M2^{xii}—Co6—Mn5^{xii}	65.707(10)
M1^{ix}—Mn5—M2^{ix}	98.158(10)	M2^{xiii}—Co6—Mn5^{xii}	117.623(14)
M1—Mn5—M2^{ix}	120.021(14)	Ge2—Co6—Mn5^{xii}	65.231(9)
Co6^{vi}—Mn5—M2^{ix}	49.596(8)	Ge1—Co6—Mn5^{xii}	112.512(10)
Co6^{vii}—Mn5—M2^{ix}	96.031(13)	M1^{xiii}—Co6—Mn5^{xii}	114.191(14)
M3^{ix}—Mn5—M2^{ix}	116.438(10)	M1^{xii}—Co6—Mn5^{xii}	62.104(10)
M3—Mn5—M2^{ix}	143.712(15)	Mn2^{viii}—Co6—Mn5^{xii}	64.224(9)
Ge2^{vii}—Mn5—M2^{ix}	145.485(11)	Mn6^{viii}—Co6—Mn5^{xii}	117.008(9)
Ge2^{vi}—Mn5—M2^{ix}	94.371(8)	M2^{xii}—Co6—Mn5^{xvi}	117.623(14)
M1^{ix}—Mn5—M2	120.022(14)	M2^{xiii}—Co6—Mn5^{xvi}	65.707(10)
M1—Mn5—M2	98.158(10)	Ge2—Co6—Mn5^{xvi}	65.231(9)
Co6^{vi}—Mn5—M2	96.031(13)	Ge1—Co6—Mn5^{xvi}	112.512(10)
Co6^{vii}—Mn5—M2	49.596(8)	M1^{xiii}—Co6—Mn5^{xvi}	62.104(10)
M3^{ix}—Mn5—M2	143.712(15)	M1^{xii}—Co6—Mn5^{xvi}	114.191(14)
M3—Mn5—M2	116.437(10)	Mn2^{viii}—Co6—Mn5^{xvi}	64.224(9)
Ge2^{vii}—Mn5—M2	94.372(8)	Mn6^{viii}—Co6—Mn5^{xvi}	117.008(9)
Ge2^{vi}—Mn5—M2	145.485(11)	Mn5^{xii}—Co6—Mn5^{xvi}	119.767(17)
M2^{ix}—Mn5—M2	51.621(11)	M2^{xii}—Co6—Mn3^{xvi}	117.340(14)

$M3^{ix}$—Mn6—M3	49.887(11)	$M2^{xiii}$—Co6—$Mn3^{xvi}$	65.657(10)
$M3^{ix}$—Mn6—$M3^{x}$	115.825(13)	Ge2—Co6—$Mn3^{xvi}$	112.92(1)
M3—Mn6—$M3^{x}$	94.790(11)	Ge1—Co6—$Mn3^{xvi}$	65.014(9)
$M3^{ix}$—Mn6—$M3^{viii}$	94.791(11)	$M1^{xiii}$—Co6—$Mn3^{xvi}$	62.771(10)
M3—Mn6—$M3^{viii}$	115.825(13)	$M1^{xii}$—Co6—$Mn3^{xvi}$	114.613(14)
$M3^{x}$—Mn6—$M3^{viii}$	49.349(11)	$Mn2^{viii}$—Co6—$Mn3^{xvi}$	117.784(9)
$M3^{ix}$—Mn6—$Co4^{iv}$	53.679(8)	$Mn6^{viii}$—Co6—$Mn3^{xvi}$	64.403(9)
M3—Mn6—$Co4^{iv}$	98.022(13)	$Mn5^{xii}$—Co6—$Mn3^{xvi}$	176.675(16)
$M3^{x}$—Mn6—$Co4^{iv}$	143.553(11)	$Mn5^{xvi}$—Co6—$Mn3^{xvi}$	60.529(10)
$M3^{viii}$—Mn6—$Co4^{iv}$	94.585(9)	$M2^{xii}$—Co6—$Mn3^{xii}$	65.657(10)
$M3^{ix}$—Mn6—$Co4^{iii}$	98.022(13)	$M2^{xiii}$—Co6—$Mn3^{xii}$	117.340(14)
M3—Mn6—$Co4^{iii}$	53.679(8)	Ge2—Co6—$Mn3^{xii}$	112.92(1)
$M3^{x}$—Mn6—$Co4^{iii}$	94.585(9)	Ge1—Co6—$Mn3^{xii}$	65.014(9)
$M3^{viii}$—Mn6—$Co4^{iii}$	143.553(11)	$M1^{xiii}$—Co6—$Mn3^{xii}$	114.613(14)
$Co4^{iv}$—Mn6—$Co4^{iii}$	120.386(17)	$M1^{xii}$—Co6—$Mn3^{xii}$	62.771(10)
$M3^{ix}$—Mn6—$Co6^{viii}$	146.220(11)	$Mn2^{viii}$—Co6—$Mn3^{xii}$	117.784(9)
M3—Mn6—$Co6^{viii}$	146.221(11)	$Mn6^{viii}$—Co6—$Mn3^{xii}$	64.403(9)
$M3^{x}$—Mn6—$Co6^{viii}$	94.715(13)	$Mn5^{xii}$—Co6—$Mn3^{xii}$	60.529(10)
$M3^{viii}$—Mn6—$Co6^{viii}$	94.715(13)	$Mn5^{xvi}$—Co6—$Mn3^{xii}$	176.675(16)
$Co4^{iv}$—Mn6—$Co6^{viii}$	93.274(11)	$Mn3^{xvi}$—Co6—$Mn3^{xii}$	118.958(17)
$Co4^{iii}$—Mn6—$Co6^{viii}$	93.274(11)	M3—Ge1—$M3^{ix}$	59.156(12)
$M3^{ix}$—Mn6—$M2^{iv}$	97.42(1)	M3—Ge1—Co6	118.982(12)
M3—Mn6—$M2^{iv}$	119.110(13)	$M3^{ix}$—Ge1—Co6	118.983(12)
$M3^{x}$—Mn6—$M2^{iv}$	143.508(15)	M3—Ge1—$M1^{xiii}$	177.501(10)
$M3^{viii}$—Mn6—$M2^{iv}$	116.758(10)	$M3^{ix}$—Ge1—$M1^{xiii}$	118.691(8)
$Co4^{iv}$—Mn6—$M2^{iv}$	49.922(8)	Co6—Ge1—$M1^{xiii}$	62.922(9)
$Co4^{iii}$—Mn6—$M2^{iv}$	95.244(13)	M3—Ge1—$M1^{xii}$	118.690(8)
$Co6^{viii}$—Mn6—$M2^{iv}$	49.700(9)	$M3^{ix}$—Ge1—$M1^{xii}$	177.50(1)
$M3^{ix}$—Mn6—$M2^{v}$	119.109(13)	Co6—Ge1—$M1^{xii}$	62.922(10)
M3—Mn6—$M2^{v}$	97.421(10)	$M1^{xiii}$—Ge1—$M1^{xii}$	63.430(12)
$M3^{x}$—Mn6—$M2^{v}$	116.758(10)	M3—Ge1—Co5	119.905(12)
$M3^{viii}$—Mn6—$M2^{v}$	143.508(15)	$M3^{ix}$—Ge1—Co5	119.904(12)
$Co4^{iv}$—Mn6—$M2^{v}$	95.243(13)	Co6—Ge1—Co5	111.164(13)
$Co4^{iii}$—Mn6—$M2^{v}$	49.922(8)	$M1^{xiii}$—Ge1—Co5	59.614(10)

Co6^{viii}—Mn6—M2^{v}	49.700(9)	M1^{xii}—Ge1—Co5	59.614(10)
M2^{iv}—Mn6—M2^{v}	50.131(11)	M3—Ge1—Mn2^{xvii}	114.197(12)
M3^{ix}—Mn6—Mn4	60.644(11)	M3^{ix}—Ge1—Mn2^{xvii}	63.868(10)
M3—Mn6—Mn4	60.645(11)	Co6—Ge1—Mn2^{xvii}	115.560(9)
M3^{x}—Mn6—Mn4	151.676(9)	M1^{xiii}—Ge1—Mn2^{xvii}	63.308(10)
M3^{viii}—Mn6—Mn4	151.676(9)	M1^{xii}—Ge1—Mn2^{xvii}	117.153(12)
Co4^{iv}—Mn6—Mn4	60.226(8)	Co5—Ge1—Mn2^{xvii}	66.048(8)
Co4^{iii}—Mn6—Mn4	60.226(9)	M3—Ge1—Mn2^{xviii}	63.868(10)
Co6^{viii}—Mn6—Mn4	99.175(15)	M3^{ix}—Ge1—Mn2^{xviii}	114.196(13)
M2^{iv}—Mn6—Mn4	58.470(11)	Co6—Ge1—Mn2^{xviii}	115.560(9)
M2^{v}—Mn6—Mn4	58.470(11)	M1^{xiii}—Ge1—Mn2^{xviii}	117.153(12)
M3^{ix}—Mn6—Ge1^{viii}	143.795(12)	M1^{xii}—Ge1—Mn2^{xviii}	63.308(10)
M3—Mn6—Ge1^{viii}	143.795(12)	Co5—Ge1—Mn2^{xviii}	66.048(9)
M3^{x}—Mn6—Ge1^{viii}	49.600(8)	Mn2^{xvii}—Ge1—Mn2^{xviii}	118.929(16)
M3^{viii}—Mn6—Ge1^{viii}	49.600(8)	M3—Ge1—Mn3^{xii}	65.413(10)
Co4^{iv}—Mn6—Ge1^{viii}	114.724(9)	M3^{ix}—Ge1—Mn3^{xii}	115.463(12)
Co4^{iii}—Mn6—Ge1^{viii}	114.724(9)	Co6—Ge1—Mn3^{xii}	64.381(8)
Co6^{viii}—Mn6—Ge1^{viii}	50.690(11)	M1^{xiii}—Ge1—Mn3^{xii}	117.081(12)
M2^{iv}—Mn6—Ge1^{viii}	94.643(12)	M1^{xii}—Ge1—Mn3^{xii}	63.529(10)
M2^{v}—Mn6—Ge1^{viii}	94.643(12)	Co5—Ge1—Mn3^{xii}	114.688(9)
Mn4—Mn6—Ge1^{viii}	149.866(16)	Mn2^{xvii}—Ge1—Mn3^{xii}	179.256(13)
Co5—Mn7—Co5^{xi}	119.090(15)	Mn2^{xviii}—Ge1—Mn3^{xii}	61.556(10)
Co5—Mn7—Mn7^{xi}	59.937(15)	M3—Ge1—Mn3^{xvi}	115.463(13)
Co5^{xi}—Mn7—Mn7^{xi}	59.153(14)	M3^{ix}—Ge1—Mn3^{xvi}	65.414(10)
Co5—Mn7—M1^{xii}	55.019(10)	Co6—Ge1—Mn3^{xvi}	64.381(9)
Co5^{xi}—Mn7—M1^{xii}	149.038(8)	M1^{xiii}—Ge1—Mn3^{xvi}	63.529(10)
Mn7^{xi}—Mn7—M1^{xii}	106.620(16)	M1^{xii}—Ge1—Mn3^{xvi}	117.081(12)
Co5—Mn7—M1^{xiii}	55.018(10)	Co5—Ge1—Mn3^{xvi}	114.688(9)
Co5^{xi}—Mn7—M1^{xiii}	149.038(8)	Mn2^{xvii}—Ge1—Mn3^{xvi}	61.556(10)
Mn7^{xi}—Mn7—M1^{xiii}	106.620(16)	Mn2^{xviii}—Ge1—Mn3^{xvi}	179.256(13)
M1^{xii}—Mn7—M1^{xiii}	57.676(12)	Mn3^{xii}—Ge1—Mn3^{xvi}	117.953(16)
Co5—Mn7—Co4^{xi}	148.264(17)	M3—Ge1—Mn6^{viii}	64.211(10)
Co5^{xi}—Mn7—Co4^{xi}	92.646(14)	M3^{ix}—Ge1—Mn6^{viii}	64.212(10)
Mn7^{xi}—Mn7—Co4^{xi}	151.80(2)	Co6—Ge1—Mn6^{viii}	63.874(12)

$M1^{xii}$—Mn7—$Co4^{xi}$	98.002(12)	$M1^{xiii}$—Ge1—$Mn6^{viii}$	116.408(12)
$M1^{xiii}$—Mn7—$Co4^{xi}$	98.002(12)	$M1^{xii}$—Ge1—$Mn6^{viii}$	116.408(12)
Co5—Mn7—Ge2	95.736(15)	Co5—Ge1—$Mn6^{viii}$	175.038(14)
$Co5^{xi}$—Mn7—Ge2	145.174(16)	$Mn2^{xvii}$—Ge1—$Mn6^{viii}$	115.523(8)
$Mn7^{xi}$—Mn7—Ge2	155.67(2)	$Mn2^{xviii}$—Ge1—$Mn6^{viii}$	115.523(9)
$M1^{xii}$—Mn7—Ge2	53.008(9)	$Mn3^{xii}$—Ge1—$Mn6^{viii}$	63.754(8)
$M1^{xiii}$—Mn7—Ge2	53.008(9)	$Mn3^{xvi}$—Ge1—$Mn6^{viii}$	63.754(8)
$Co4^{xi}$—Mn7—Ge2	52.528(11)	Co6—Ge2—$M3^{xii}$	119.262(11)
Co5—Mn7—$M2^{xi}$	150.333(9)	Co6—Ge2—$M3^{xiii}$	119.262(11)
$Co5^{xi}$—Mn7—$M2^{xi}$	52.274(10)	$M3^{xii}$—Ge2—$M3^{xiii}$	63.317(13)
$Mn7^{xi}$—Mn7—$M2^{xi}$	104.340(17)	Co6—Ge2—$Co4^{xi}$	177.355(15)
$M1^{xii}$—Mn7—$M2^{xi}$	148.909(15)	$M3^{xii}$—Ge2—$Co4^{xi}$	62.862(9)
$M1^{xiii}$—Mn7—$M2^{xi}$	115.285(9)	$M3^{xiii}$—Ge2—$Co4^{xi}$	62.862(9)
$Co4^{xi}$—Mn7—$M2^{xi}$	51.319(9)	Co6—Ge2—$M1^{xiii}$	63.612(9)
Ge2—Mn7—$M2^{xi}$	97.402(13)	$M3^{xii}$—Ge2—$M1^{xiii}$	177.092(13)
Co5—Mn7—$M2^{xiv}$	150.333(9)	$M3^{xiii}$—Ge2—$M1^{xiii}$	116.081(9)
$Co5^{xi}$—Mn7—$M2^{xiv}$	52.274(10)	$Co4^{xi}$—Ge2—$M1^{xiii}$	114.252(11)
$Mn7^{xi}$—Mn7—$M2^{xiv}$	104.340(17)	Co6—Ge2—$M1^{xii}$	63.612(9)
$M1^{xii}$—Mn7—$M2^{xiv}$	115.285(10)	$M3^{xii}$—Ge2—$M1^{xii}$	116.081(9)
$M1^{xiii}$—Mn7—$M2^{xiv}$	148.909(15)	$M3^{xiii}$—Ge2—$M1^{xii}$	177.092(13)
$Co4^{xi}$—Mn7—$M2^{xiv}$	51.319(9)	$Co4^{xi}$—Ge2—$M1^{xii}$	114.252(11)
Ge2—Mn7—$M2^{xiv}$	97.402(13)	$M1^{xiii}$—Ge2—$M1^{xii}$	64.363(13)
$M2^{xi}$—Mn7—$M2^{xiv}$	52.842(11)	Co6—Ge2—$Mn2^{viii}$	65.009(13)
Co5—Mn7—$Mn4^{xii}$	107.205(11)	$M3^{xii}$—Ge2—$Mn2^{viii}$	64.843(11)
$Co5^{xi}$—Mn7—$Mn4^{xii}$	105.200(11)	$M3^{xiii}$—Ge2—$Mn2^{viii}$	64.843(10)
$Mn7^{xi}$—Mn7—$Mn4^{xii}$	123.386(9)	$Co4^{xi}$—Ge2—$Mn2^{viii}$	117.636(14)
$M1^{xii}$—Mn7—$Mn4^{xii}$	57.268(9)	$M1^{xiii}$—Ge2—$Mn2^{viii}$	117.714(12)
$M1^{xiii}$—Mn7—$Mn4^{xii}$	105.334(13)	$M1^{xii}$—Ge2—$Mn2^{viii}$	117.714(12)
$Co4^{xi}$—Mn7—$Mn4^{xii}$	60.267(9)	Co6—Ge2—Mn7	114.484(14)
Ge2—Mn7—$Mn4^{xii}$	60.538(9)	$M3^{xii}$—Ge2—Mn7	115.570(12)
$M2^{xi}$—Mn7—$Mn4^{xii}$	102.420(13)	$M3^{xiii}$—Ge2—Mn7	115.570(12)
$M2^{xiv}$—Mn7—$Mn4^{xii}$	58.15(1)	$Co4^{xi}$—Ge2—Mn7	62.872(12)
Co5—Mn7—$Mn4^{xvi}$	107.205(11)	$M1^{xiii}$—Ge2—Mn7	61.881(10)
$Co5^{xi}$—Mn7—$Mn4^{xvi}$	105.200(11)	$M1^{xii}$—Ge2—Mn7	61.881(10)

Mn7^{xi}—Mn7—Mn4^{xvi}	123.386(9)	Mn2^{viii}—Ge2—Mn7	179.493(13)
M1^{xii}—Mn7—Mn4^{xvi}	105.334(13)	Co6—Ge2—Mn5^{xii}	64.342(8)
M1^{xiii}—Mn7—Mn4^{xvi}	57.268(9)	M3^{xii}—Ge2—Mn5^{xii}	64.529(9)
Co4^{xi}—Mn7—Mn4^{xvi}	60.267(9)	M3^{xiii}—Ge2—Mn5^{xii}	118.121(13)
Ge2—Mn7—Mn4^{xvi}	60.538(9)	Co4^{xi}—Ge2—Mn5^{xii}	116.436(8)
M2^{xi}—Mn7—Mn4^{xvi}	58.150(9)	M1^{xiii}—Ge2—Mn5^{xii}	117.538(12)
M2^{xiv}—Mn7—Mn4^{xvi}	102.420(13)	M1^{xii}—Ge2—Mn5^{xii}	63.105(10)
Mn4^{xii}—Mn7—Mn4^{xvi}	113.160(17)	Mn2^{viii}—Ge2—Mn5^{xii}	64.445(8)
Co5—Mn7—Mn1^{xvii}	56.266(8)	Mn7—Ge2—Mn5^{xii}	115.399(9)
Co5^{xi}—Mn7—Mn1^{xvii}	96.518(11)	Co6—Ge2—Mn5^{xvi}	64.342(8)
Mn7^{xi}—Mn7—Mn1^{xvii}	64.331(10)	M3^{xii}—Ge2—Mn5^{xvi}	118.121(12)
M1^{xii}—Mn7—Mn1^{xvii}	101.065(13)	M3^{xiii}—Ge2—Mn5^{xvi}	64.529(9)
M1^{xiii}—Mn7—Mn1^{xvii}	53.737(9)	Co4^{xi}—Ge2—Mn5^{xvi}	116.436(9)
Co4^{xi}—Mn7—Mn1^{xvii}	124.506(8)	M1^{xiii}—Ge2—Mn5^{xvi}	63.105(10)
Ge2—Mn7—Mn1^{xvii}	103.513(11)	M1^{xii}—Ge2—Mn5^{xvi}	117.538(12)
M2^{xi}—Mn7—Mn1^{xvii}	94.725(9)	Mn2^{viii}—Ge2—Mn5^{xvi}	64.445(9)
M2^{xiv}—Mn7—Mn1^{xvii}	143.619(13)	Mn7—Ge2—Mn5^{xvi}	115.399(9)
Mn4^{xii}—Mn7—Mn1^{xvii}	157.713(16)	Mn5^{xii}—Ge2—Mn5^{xvi}	118.342(15)
Mn4^{xvi}—Mn7—Mn1^{xvii}	64.509(10)	Co6—Ge2—Mn4^{xvi}	114.905(9)
M1^{ix}—M1—Ge2^{vii}	122.180(6)	M3^{xii}—Ge2—Mn4^{xvi}	117.402(12)
M1^{ix}—M1—Co5^{vii}	121.821(7)	M3^{xiii}—Ge2—Mn4^{xvi}	64.375(10)
Ge2^{vii}—M1—Co5^{vii}	109.870(11)	Co4^{xi}—Ge2—Mn4^{xvi}	64.201(9)
M1^{ix}—M1—Ge1^{vii}	121.716(6)	M1^{xiii}—Ge2—Mn4^{xvi}	60.469(9)
Ge2^{vii}—M1—Ge1^{vii}	104.617(10)	M1^{xii}—Ge2—Mn4^{xvi}	114.394(13)
Co5^{vii}—M1—Ge1^{vii}	60.484(11)	Mn2^{viii}—Ge2—Mn4^{xvi}	117.625(9)
M1^{ix}—M1—Co6^{vii}	120.484(6)	Mn7—Ge2—Mn4^{xvi}	62.518(9)
Ge2^{vii}—M1—Co6^{vii}	57.82(1)	Mn5^{xii}—Ge2—Mn4^{xvi}	177.498(13)
Co5^{vii}—M1—Co6^{vii}	107.755(11)	Mn5^{xvi}—Ge2—Mn4^{xvi}	62.516(10)
Ge1^{vii}—M1—Co6^{vii}	57.844(11)	Co6—Ge2—Mn4^{xii}	114.905(9)
M1^{ix}—M1—Mn1^{i}	63.736(6)	M3^{xii}—Ge2—Mn4^{xii}	64.375(10)
Ge2^{vii}—M1—Mn1^{i}	129.042(13)	M3^{xiii}—Ge2—Mn4^{xii}	117.402(12)
Co5^{vii}—M1—Mn1^{i}	64.080(9)	Co4^{xi}—Ge2—Mn4^{xii}	64.201(8)
Ge1^{vii}—M1—Mn1^{i}	112.246(13)	M1^{xiii}—Ge2—Mn4^{xii}	114.394(13)
Co6^{vii}—M1—Mn1^{i}	170.084(13)	M1^{xii}—Ge2—Mn4^{xii}	60.469(9)

$M1^{ix}$—M1—$M1^{xv}$	180.000	$Mn2^{viii}$—Ge2—$Mn4^{xii}$	117.625(9)
$Ge2^{vii}$—M1—$M1^{xv}$	57.818(6)	Mn7—Ge2—$Mn4^{xii}$	62.518(8)
$Co5^{vii}$—M1—$M1^{xv}$	58.180(7)	$Mn5^{xii}$—Ge2—$Mn4^{xii}$	62.516(10)
$Ge1^{vii}$—M1—$M1^{xv}$	58.285(6)	$Mn5^{xvi}$—Ge2—$Mn4^{xii}$	177.498(13)
$Co6^{vii}$—M1—$M1^{xv}$	59.515(6)	$Mn4^{xvi}$—Ge2—$Mn4^{xii}$	116.520(15)
$Mn1^{i}$—M1—$M1^{xv}$	116.265(6)	$M3^{xvii}$—Ge3—$M3^{vii}$	180.000(11)
$M1^{ix}$—M1—$Mn7^{vii}$	118.837(6)	$M3^{xvii}$—Ge3—$M3^{xx}$	116.701(11)
$Ge2^{vii}$—M1—$Mn7^{vii}$	65.11(1)	$M3^{vii}$—Ge3—$M3^{xx}$	63.299(11)
$Co5^{vii}$—M1—$Mn7^{vii}$	61.391(11)	$M3^{xvii}$—Ge3—$M3^{xix}$	63.299(11)
$Ge1^{vii}$—M1—$Mn7^{vii}$	110.953(11)	$M3^{vii}$—Ge3—$M3^{xix}$	116.701(11)
$Co6^{vii}$—M1—$Mn7^{vii}$	112.468(11)	$M3^{xx}$—Ge3—$M3^{xix}$	180.000(11)
$Mn1^{i}$—M1—$Mn7^{vii}$	69.452(10)	$M3^{xvii}$—Ge3—Co4	62.879(8)
$M1^{xv}$—M1—$Mn7^{vii}$	61.162(6)	$M3^{vii}$—Ge3—Co4	117.121(8)
$M1^{ix}$—M1—Mn4	64.869(6)	$M3^{xx}$—Ge3—Co4	117.121(8)
$Ge2^{vii}$—M1—Mn4	67.822(9)	$M3^{xix}$—Ge3—Co4	62.879(8)
$Co5^{vii}$—M1—Mn4	120.509(13)	$M3^{xvii}$—Ge3—$Co4^{xxi}$	117.120(8)
$Ge1^{vii}$—M1—Mn4	172.407(11)	$M3^{vii}$—Ge3—$Co4^{xxi}$	62.880(8)
$Co6^{vii}$—M1—Mn4	116.276(13)	$M3^{xx}$—Ge3—$Co4^{xxi}$	62.880(8)
$Mn1^{i}$—M1—Mn4	73.558(13)	$M3^{xix}$—Ge3—$Co4^{xxi}$	117.120(8)
$M1^{xv}$—M1—Mn4	115.129(6)	Co4—Ge3—$Co4^{xxi}$	180.000
$Mn7^{vii}$—M1—Mn4	65.811(10)	$M3^{xvii}$—Ge3—$Mn6^{vi}$	63.733(9)
$M1^{ix}$—M1—Mn5	65.694(6)	$M3^{vii}$—Ge3—$Mn6^{vi}$	116.267(9)
$Ge2^{vii}$—M1—Mn5	65.659(9)	$M3^{xx}$—Ge3—$Mn6^{vi}$	62.997(9)
$Co5^{vii}$—M1—Mn5	171.409(11)	$M3^{xix}$—Ge3—$Mn6^{vi}$	117.003(9)
$Ge1^{vii}$—M1—Mn5	112.691(12)	Co4—Ge3—$Mn6^{vi}$	116.262(7)
$Co6^{vii}$—M1—Mn5	63.679(9)	$Co4^{xxi}$—Ge3—$Mn6^{vi}$	63.737(7)
$Mn1^{i}$—M1—Mn5	124.506(11)	$M3^{xvii}$—Ge3—$Mn6^{xviii}$	116.267(9)
$M1^{xv}$—M1—Mn5	114.305(6)	$M3^{vii}$—Ge3—$Mn6^{xviii}$	63.733(9)
$Mn7^{vii}$—M1—Mn5	120.081(12)	$M3^{xx}$—Ge3—$Mn6^{xviii}$	117.003(9)
Mn4—M1—Mn5	65.484(12)	$M3^{xix}$—Ge3—$Mn6^{xviii}$	62.997(9)
$M1^{ix}$—M1—$Mn2^{i}$	65.842(6)	Co4—Ge3—$Mn6^{xviii}$	63.738(7)
$Ge2^{vii}$—M1—$Mn2^{i}$	169.331(11)	$Co4^{xxi}$—Ge3—$Mn6^{xviii}$	116.263(7)
$Co5^{vii}$—M1—$Mn2^{i}$	66.896(11)	$Mn6^{vi}$—Ge3—$Mn6^{xviii}$	180.000(18)
$Ge1^{vii}$—M1—$Mn2^{i}$	64.798(9)	$M3^{xvii}$—Ge3—$Mn6^{xvii}$	62.997(9)

Co6^{vii}—M1—Mn2^{i}	112.689(13)	M3^{vii}—Ge3—Mn6^{xvii}	117.003(9)
Mn1^{i}—M1—Mn2^{i}	59.687(12)	M3^{xx}—Ge3—Mn6^{xvii}	63.733(9)
M1^{xv}—M1—Mn2^{i}	114.160(5)	M3^{xix}—Ge3—Mn6^{xvii}	116.267(9)
Mn7^{vii}—M1—Mn2^{i}	118.741(12)	Co4—Ge3—Mn6^{xvii}	63.738(7)
Mn4—M1—Mn2^{i}	122.778(11)	Co4^{xxi}—Ge3—Mn6^{xvii}	116.263(7)
Mn5—M1—Mn2^{i}	116.052(11)	Mn6^{vi}—Ge3—Mn6^{xvii}	62.626(13)
M1^{ix}—M1—Mn3	66.002(5)	Mn6^{xviii}—Ge3—Mn6^{xvii}	117.374(13)
Ge2^{vii}—M1—Mn3	112.894(11)	M3^{xvii}—Ge3—Mn6^{vii}	117.003(9)
Co5^{vii}—M1—Mn3	116.565(13)	M3^{vii}—Ge3—Mn6^{vii}	62.997(9)
Ge1^{vii}—M1—Mn3	64.889(9)	M3^{xx}—Ge3—Mn6^{vii}	116.267(9)
Co6^{vii}—M1—Mn3	63.475(9)	M3^{xix}—Ge3—Mn6^{vii}	63.733(9)
Mn1^{i}—M1—Mn3	114.054(11)	Co4—Ge3—Mn6^{vii}	116.262(7)
M1^{xv}—M1—Mn3	113.999(5)	Co4^{xxi}—Ge3—Mn6^{vii}	63.737(7)
Mn7^{vii}—M1—Mn3	175.160(9)	Mn6^{vi}—Ge3—Mn6^{vii}	117.374(13)
Mn4—M1—Mn3	117.981(11)	Mn6^{xviii}—Ge3—Mn6^{vii}	62.626(13)
Mn5—M1—Mn3	61.207(11)	Mn6^{xvii}—Ge3—Mn6^{vii}	180.000(18)
Mn2^{i}—M1—Mn3	62.387(11)	M3^{xvii}—Ge3—Mn3	115.316(9)
Co6^{vii}—M2—Co4	118.573(14)	M3^{vii}—Ge3—Mn3	64.684(9)
Co6^{vii}—M2—Co5	120.310(13)	M3^{xx}—Ge3—Mn3	64.684(9)
Co4—M2—Co5	108.778(11)	M3^{xix}—Ge3—Mn3	115.316(9)
Co6^{vii}—M2—M2^{xv}	59.614(6)	Co4—Ge3—Mn3	62.532(10)
Co4—M2—M2^{xv}	121.291(6)	Co4^{xxi}—Ge3—Mn3	117.469(10)
Co5—M2—M2^{xv}	121.096(6)	Mn6^{vi}—Ge3—Mn3	116.485(7)
Co6^{vii}—M2—M2^{ix}	120.385(7)	Mn6^{xviii}—Ge3—Mn3	63.515(7)
Co4—M2—M2^{ix}	58.708(6)	Mn6^{xvii}—Ge3—Mn3	63.515(7)
Co5—M2—M2^{ix}	58.905(6)	Mn6^{vii}—Ge3—Mn3	116.485(7)
M2^{xv}—M2—M2^{ix}	179.999(17)	M3^{xvii}—Ge3—Mn3^{xxi}	64.683(9)
Co6^{vii}—M2—Mn1^{xviii}	109.476(11)	M3^{vii}—Ge3—Mn3^{xxi}	115.317(9)
Co4—M2—Mn1^{xviii}	124.958(13)	M3^{xx}—Ge3—Mn3^{xxi}	115.316(9)
Co5—M2—Mn1^{xviii}	63.309(10)	M3^{xix}—Ge3—Mn3^{xxi}	64.684(9)
M2^{xv}—M2—Mn1^{xviii}	63.402(6)	Co4—Ge3—Mn3^{xxi}	117.467(10)
M2^{ix}—M2—Mn1^{xviii}	116.599(6)	Co4^{xxi}—Ge3—Mn3^{xxi}	62.532(10)
Co6^{vii}—M2—Mn4^{xviii}	113.425(11)	Mn6^{vi}—Ge3—Mn3^{xxi}	63.516(7)
Co4—M2—Mn4^{xviii}	66.261(9)	Mn6^{xviii}—Ge3—Mn3^{xxi}	116.484(7)

Co5—M2—Mn4^{xviii}	117.773(12)	Mn6^{xvii}—Ge3—Mn3^{xxi}	116.484(7)
M2^{xv}—M2—Mn4^{xviii}	64.343(5)	Mn6^{vii}—Ge3—Mn3^{xxi}	63.516(7)
M2^{ix}—M2—Mn4^{xviii}	115.657(6)	Mn3—Ge3—Mn3^{xxi}	180.000
Mn1^{xviii}—M2—Mn4^{xviii}	71.342(11)		

(i) -x, 1-y, -z; (ii) -x, 1-y, z; (iii) 0.5-x, 0.5+y, 0.5+z; (iv) 0.5-x, 0.5+y, -0.5+z; (v) 0.5-x, 0.5+y, 0.5-z; (vi) -0.5+x, 0.5-y, -0.5-z; (vii) -0.5+x, 0.5-y, 0.5-z; (viii) 1-x, 1-y, -z; (ix) x, y, -z; (x) 1-x, 1-y, z; (xi) 1-x, -y, -z; (xii) 0.5+x, 0.5-y, 0.5-z; (xiii) 0.5+x, 0.5-y, -0.5+z; (xiv) 1-x, -y, z; (xv) x, y, 1-z; (xvi) 0.5+x, 0.5-y, -0.5-z; (xvii) 0.5-x, -0.5+y, -0.5+z; (xviii) 0.5-x, -0.5+y, 0.5+z; (xix) 0.5-x, -0.5+y, 0.5-z; (xx) -0.5+x, 0.5-y, -0.5+z; (xxi) -x, -y, -z.

Anisotropic displacement parameters for $Mn_{14}Co_{16.2}Ge_{6.8}$, in Å^2						
**Atom**	U_{11}	U_{22}	U_{33}	U_{12}	U_{13}	U_{23}
Mn1	0.00800(13)	0.00413(12)	0.00638(12)	-0.00091(10)	0.00000	0.00000
Mn2	0.00647(12)	0.00426(12)	0.00587(12)	-0.00057(9)	0.00000	0.00000
Mn3	0.00725(13)	0.00470(12)	0.00587(12)	0.00014(9)	0.00000	0.00000
Mn4	0.00942(13)	0.00443(12)	0.00482(12)	-0.0010(1)	0.00000	0.00000
Mn5	0.00678(12)	0.00489(12)	0.00532(12)	-0.00009(10)	0.00000	0.00000
Mn6	0.00678(12)	0.00466(12)	0.00486(12)	0.00001(9)	0.00000	0.00000
Mn7	0.00578(12)	0.00354(12)	0.00787(13)	-0.00016(9)	0.00000	0.00000
Co1	0.00550(8)	0.00446(8)	0.00218(7)	-0.00012(6)	0.00007(6)	0.00049(5)
*M*2	0.00668(8)	0.00436(8)	0.00367(8)	-0.00072(5)	0.00102(5)	-0.00110(5)
Co3	0.00642(8)	0.00375(8)	0.00233(7)	0.00010(6)	0.00082(6)	-0.00088(5)
Co4	0.00513(11)	0.00262(10)	0.00599(11)	-0.00082(8)	0.00000	0.00000
Co5	0.00484(11)	0.00480(11)	0.00539(11)	-0.00129(8)	0.00000	0.00000
Co6	0.00573(11)	0.00276(10)	0.00596(11)	-0.00068(8)	0.00000	0.00000
Ge1	0.00619(9)	0.00331(9)	0.00516(9)	0.00175(7)	0.00000	0.00000
Ge2	0.00476(9)	0.00358(9)	0.00582(9)	0.00096(7)	0.00000	0.00000
Ge3	0.00497(12)	0.00412(12)	0.00515(12)	0.00154(9)	0.00000	0.00000

*M*2 = 0.54Co2+0.46Ge22

Selected geometric informations for $Mn_{14}Co_{16.2}Ge_{6.8}$

Atoms 1,2	d 1,2 [Å]	Atoms 1,2	d 1,2 [Å]
Mn1—Mn1^{i}	2.3329(8)	Mn5—Co6^{vii}	2.7936(3)
Mn1—Co1^{ii}	2.5613(5)	Mn5—Co3^{ix}	2.8042(5)
Mn1—Co1^{i}	2.5613(5)	Mn5—Co3	2.8042(5)
Mn1—Co5^{iii}	2.6523(3)	Mn5—Ge2^{vii}	2.8157(3)
Mn1—Co5^{iv}	2.6523(3)	Mn5—Ge2^{vi}	2.8157(3)
Mn1—M2^{v}	2.6589(5)	Mn5—M2	2.8214(5)
Mn1—M2^{iv}	2.6589(5)	Mn5—M2^{ix}	2.8215(5)
Mn1—Mn2	2.6611(6)	Mn6—Co3^{ix}	2.7587(5)
Mn1—Mn7^{iv}	2.9736(4)	Mn6—Co3	2.7587(5)
Mn1—Mn7^{iii}	2.9736(4)	Mn6—Co3^{xi}	2.7800(5)

Mn1—Mn7^{vi}	3.0135(4)	Mn6—Co3^{viii}	2.7800(5)
Mn1—Mn7^{vii}	3.0135(4)	Mn6—Co4^{iv}	2.7848(3)
Mn2—Ge2^{viii}	2.7233(5)	Mn6—Co4^{iii}	2.7848(3)
Mn2—Co6^{viii}	2.7587(6)	Mn6—Co6^{viii}	2.7942(6)
Mn2—Co3^{v}	2.7591(5)	Mn6—M2^{iv}	2.8084(5)
Mn2—Co3^{iv}	2.7591(5)	Mn6—M2^{v}	2.8084(5)
Mn2—Co1^{i}	2.7684(5)	Mn6—Ge3^{xii}	2.8288(3)
Mn2—Co1^{ii}	2.7684(5)	Mn6—Ge3^{xiii}	2.8288(3)
Mn2—Ge1^{iv}	2.8055(3)	Mn7—Co5	2.6054(5)
Mn2—Ge1^{iii}	2.8055(3)	Mn7—Co5^{xiv}	2.6320(6)
Mn2—M2^{iv}	2.8597(5)	Mn7—Mn7^{xiv}	2.6529(8)
Mn2—M2^{v}	2.8597(5)	Mn7—Co1^{xii}	2.6628(5)
Mn2—Mn3^{i}	2.8791(6)	Mn7—Co1^{xv}	2.6628(5)
Mn3—Co4	2.7484(6)	Mn7—Co4^{xiv}	2.6935(6)
Mn3—Co1^{ix}	2.7916(5)	Mn7—Ge2	2.7374(5)
Mn3—Co1	2.7916(5)	Mn7—M2^{xiv}	2.7570(4)
Mn3—Co6^{vii}	2.8050(3)	Mn7—M2^{xvi}	2.7570(4)
Mn3—Co6^{vi}	2.8050(3)	Co1—Co1^{ix}	2.2683(5)
Mn3—Mn5	2.8200(6)	Co1—Ge2^{vii}	2.4063(4)
Mn3—Co3^{x}	2.8207(5)	Co1—Co5^{vii}	2.4334(4)
Mn3—Co3^{vii}	2.8207(5)	Co1—Ge1^{vii}	2.4404(4)
Mn3—Ge1^{vi}	2.8209(3)	Co1—Co6^{vii}	2.5301(4)
Mn3—Ge1^{vii}	2.8209(3)	Co1—Co1^{xvii}	2.5655(5)
Mn3—M2^{ix}	2.8211(5)	M2—Co6^{vii}	2.3555(4)
Mn3—M2	2.8211(5)	M2—Co4	2.3640(4)
Mn4—Co1	2.6697(5)	M2—Co5	2.3733(4)
Mn4—Co1^{ix}	2.6697(5)	M2—M2^{xvii}	2.3805(5)
Mn4—M2^{v}	2.7533(5)	M2—M2^{ix}	2.4533(5)
Mn4—M2^{iv}	2.7533(5)	Co3—Co3^{ix}	2.3230(5)
Mn4—Co4^{iii}	2.8124(3)	Co3—Ge1	2.3541(4)
Mn4—Co4^{iv}	2.8124(3)	Co3—Ge2^{vii}	2.3913(4)
Mn4—Co3^{ix}	2.8139(5)	Co3—Ge3^{xii}	2.3949(3)
Mn4—Co3	2.8140(5)	Co3—Co4^{iii}	2.5028(4)
Mn4—Mn6	2.8188(6)	Co3—Co3^{xvii}	2.5108(5)

Mn4—Ge2^{vi}	2.8429(3)	Co4—Ge3	2.3982(4)
Mn4—Ge2^{vii}	2.8429(3)	Co4—Ge2^{xiv}	2.4063(5)
Mn4—Mn7^{vi}	2.8953(4)	Co5—Ge1	2.4579(5)
Mn5—Co1	2.7527(5)	Co6—Ge2	2.3922(5)
Mn5—Co1^{ix}	2.7528(5)	Co6—Ge1	2.4020(5)
Mn5—Co6^{vi}	2.7936(3)		

Atoms 1,2,3	Angle 1,2,3 [°]	Atoms 1,2,3	Angle 1,2,3 [°]
Mn1^{i}—Mn1—Co1^{ii}	115.84(2)	Co1^{xvii}—Co1—Mn3	113.971(6)
Mn1^{i}—Mn1—Co1^{i}	115.84(2)	Mn7^{vii}—Co1—Mn3	175.170(11)
Co1^{ii}—Mn1—Co1^{i}	52.568(14)	Mn4—Co1—Mn3	118.049(13)
Mn1^{i}—Mn1—Co5^{iii}	114.282(11)	Mn5—Co1—Mn3	61.140(12)
Co1^{ii}—Mn1—Co5^{iii}	55.612(11)	Mn2^{i}—Co1—Mn3	62.370(12)
Co1^{i}—Mn1—Co5^{iii}	104.019(17)	Co6^{vii}—M2—Co4	118.654(16)
Mn1^{i}—Mn1—Co5^{iv}	114.282(11)	Co6^{vii}—M2—Co5	120.187(15)
Co1^{ii}—Mn1—Co5^{iv}	104.019(17)	Co4—M2—Co5	108.793(13)
Co1^{i}—Mn1—Co5^{iv}	55.612(11)	Co6^{vii}—M2—M2^{xvii}	59.648(7)
Co5^{iii}—Mn1—Co5^{iv}	131.36(2)	Co4—M2—M2^{xvii}	121.257(7)
Mn1^{i}—Mn1—M2^{v}	115.13(2)	Co5—M2—M2^{xvii}	121.122(7)
Co1^{ii}—Mn1—M2^{v}	103.485(13)	Co6^{vii}—M2—M2^{ix}	120.352(7)
Co1^{i}—Mn1—M2^{v}	129.023(18)	Co4—M2—M2^{ix}	58.742(7)
Co5^{iii}—Mn1—M2^{v}	53.085(10)	Co5—M2—M2^{ix}	58.880(7)
Co5^{iv}—Mn1—M2^{v}	102.427(16)	M2^{xvii}—M2—M2^{ix}	180.000
Mn1^{i}—Mn1—M2^{iv}	115.13(2)	Co6^{vii}—M2—Mn1^{xix}	109.407(13)
Co1^{ii}—Mn1—M2^{iv}	129.023(18)	Co4—M2—Mn1^{xix}	124.986(15)
Co1^{i}—Mn1—M2^{iv}	103.485(13)	Co5—M2—Mn1^{xix}	63.317(11)
Co5^{iii}—Mn1—M2^{iv}	102.427(16)	M2^{xvii}—M2—Mn1^{xix}	63.407(6)
Co5^{iv}—Mn1—M2^{iv}	53.085(10)	M2^{ix}—M2—Mn1^{xix}	116.594(7)
M2^{v}—Mn1—M2^{iv}	53.185(13)	Co6^{vii}—M2—Mn4^{xix}	113.523(13)
Mn1^{i}—Mn1—Mn2	179.81(3)	Co4—M2—Mn4^{xix}	66.167(11)
Co1^{ii}—Mn1—Mn2	63.992(14)	Co5—M2—Mn4^{xix}	117.828(15)
Co1^{i}—Mn1—Mn2	63.992(14)	M2^{xvii}—M2—Mn4^{xix}	64.386(6)
Co5^{iii}—Mn1—Mn2	65.723(11)	M2^{ix}—M2—Mn4^{xix}	115.613(6)
Co5^{iv}—Mn1—Mn2	65.723(11)	Mn1^{xix}—M2—Mn4^{xix}	71.448(13)

M2^{v}—Mn1—Mn2	65.031(13)	Co6^{vii}—M2—Mn7^{xiv}	176.066(11)
M2^{iv}—Mn1—Mn2	65.031(13)	Co4—M2—Mn7^{xiv}	62.915(13)
Mn1^{i}—Mn1—Mn7^{iv}	67.972(13)	Co5—M2—Mn7^{xiv}	61.194(13)
Co1^{ii}—Mn1—Mn7^{iv}	100.030(16)	M2^{xvii}—M2—Mn7^{xiv}	116.419(6)
Co1^{i}—Mn1—Mn7^{iv}	56.928(12)	M2^{ix}—M2—Mn7^{xiv}	63.582(6)
Co5^{iii}—Mn1—Mn7^{iv}	154.839(19)	Mn1^{xix}—M2—Mn7^{xiv}	67.589(11)
Co5^{iv}—Mn1—Mn7^{iv}	54.816(11)	Mn4^{xix}—M2—Mn7^{xiv}	63.395(11)
M2^{v}—Mn1—Mn7^{iv}	150.915(13)	Co6^{vii}—M2—Mn6^{xix}	64.825(13)
M2^{iv}—Mn1—Mn7^{iv}	98.408(11)	Co4—M2—Mn6^{xix}	64.480(11)
Mn2—Mn1—Mn7^{iv}	111.936(13)	Co5—M2—Mn6^{xix}	173.246(12)
Mn1^{i}—Mn1—Mn7^{iii}	67.972(13)	M2^{xvii}—M2—Mn6^{xix}	64.924(6)
Co1^{ii}—Mn1—Mn7^{iii}	56.928(11)	M2^{ix}—M2—Mn6^{xix}	115.074(6)
Co1^{i}—Mn1—Mn7^{iii}	100.030(16)	Mn1^{xix}—M2—Mn6^{xix}	120.451(12)
Co5^{iii}—Mn1—Mn7^{iii}	54.816(11)	Mn4^{xix}—M2—Mn6^{xix}	60.894(13)
Co5^{iv}—Mn1—Mn7^{iii}	154.839(19)	Mn7^{xiv}—M2—Mn6^{xix}	114.139(14)
M2^{v}—Mn1—Mn7^{iii}	98.408(10)	Co6^{vii}—M2—Mn3	64.894(11)
M2^{iv}—Mn1—Mn7^{iii}	150.915(13)	Co4—M2—Mn3	63.297(13)
Mn2—Mn1—Mn7^{iii}	111.936(13)	Co5—M2—Mn3	113.076(13)
Mn7^{iv}—Mn1—Mn7^{iii}	108.741(19)	M2^{xvii}—M2—Mn3	115.773(6)
Mn1^{i}—Mn1—Mn7^{vi}	66.167(13)	M2^{ix}—M2—Mn3	64.226(6)
Co1^{ii}—Mn1—Mn7^{vi}	151.155(14)	Mn1^{xix}—M2—Mn3	171.344(15)
Co1^{i}—Mn1—Mn7^{vi}	99.533(11)	Mn4^{xix}—M2—Mn3	116.462(14)
Co5^{iii}—Mn1—Mn7^{vi}	152.489(19)	Mn7^{xiv}—M2—Mn3	118.397(12)
Co5^{iv}—Mn1—Mn7^{vi}	54.916(12)	Mn6^{xix}—M2—Mn3	64.032(12)
M2^{v}—Mn1—Mn7^{vi}	100.633(16)	Co6^{vii}—M2—Mn5	64.585(11)
M2^{iv}—Mn1—Mn7^{vi}	57.756(11)	Co4—M2—Mn5	111.630(13)
Mn2—Mn1—Mn7^{vi}	113.924(13)	Co5—M2—Mn5	65.478(12)
Mn7^{iv}—Mn1—Mn7^{vi}	52.600(14)	M2^{xvii}—M2—Mn5	115.771(6)
Mn7^{iii}—Mn1—Mn7^{vi}	134.139(15)	M2^{ix}—M2—Mn5	64.230(6)
Mn1^{i}—Mn1—Mn7^{vii}	66.167(13)	Mn1^{xix}—M2—Mn5	112.053(14)
Co1^{ii}—Mn1—Mn7^{vii}	99.533(11)	Mn4^{xix}—M2—Mn5	176.316(15)
Co1^{i}—Mn1—Mn7^{vii}	151.155(14)	Mn7^{xiv}—M2—Mn5	118.640(12)
Co5^{iii}—Mn1—Mn7^{vii}	54.916(12)	Mn6^{xix}—M2—Mn5	115.617(14)
Co5^{iv}—Mn1—Mn7^{vii}	152.489(19)	Mn3—M2—Mn5	59.970(12)

M2^{v}—Mn1—Mn7^{vii}	57.756(10)	Co6^{vii}—M2—Mn2^{xix}	63.005(14)
M2^{iv}—Mn1—Mn7^{vii}	100.633(16)	Co4—M2—Mn2^{xix}	173.286(11)
Mn2—Mn1—Mn7^{vii}	113.924(13)	Co5—M2—Mn2^{xix}	66.106(11)
Mn7^{iv}—Mn1—Mn7^{vii}	134.139(15)	M2^{xvii}—M2—Mn2^{xix}	65.404(6)
Mn7^{iii}—Mn1—Mn7^{vii}	52.600(14)	M2^{ix}—M2—Mn2^{xix}	114.597(6)
Mn7^{vi}—Mn1—Mn7^{vii}	106.649(18)	Mn1^{xix}—M2—Mn2^{xix}	57.522(13)
Mn1—Mn2—Ge2^{viii}	149.91(2)	Mn4^{xix}—M2—Mn2^{xix}	119.753(12)
Mn1—Mn2—Co6^{viii}	98.174(17)	Mn7^{xiv}—M2—Mn2^{xix}	115.902(15)
Ge2^{viii}—Mn2—Co6^{viii}	51.739(13)	Mn6^{xix}—M2—Mn2^{xix}	120.547(11)
Mn1—Mn2—Co3^{v}	148.954(11)	Mn3—M2—Mn2^{xix}	113.947(14)
Ge2^{viii}—Mn2—Co3^{v}	51.714(10)	Mn5—M2—Mn2^{xix}	62.636(11)
Co6^{viii}—Mn2—Co3^{v}	96.807(14)	Co3^{ix}—Co3—Ge1	60.437(7)
Mn1—Mn2—Co3^{iv}	148.954(11)	Co3^{ix}—Co3—Ge2^{vii}	121.667(7)
Ge2^{viii}—Mn2—Co3^{iv}	51.714(10)	Ge1—Co3—Ge2^{vii}	120.316(14)
Co6^{viii}—Mn2—Co3^{iv}	96.807(14)	Co3^{ix}—Co3—Ge3^{xii}	121.615(6)
Co3^{v}—Mn2—Co3^{iv}	54.130(13)	Ge1—Co3—Ge3^{xii}	121.858(14)
Mn1—Mn2—Co1^{i}	56.251(12)	Ge2^{vii}—Co3—Ge3^{xii}	105.921(11)
Ge2^{viii}—Mn2—Co1^{i}	147.504(13)	Co3^{ix}—Co3—Co4^{iii}	120.106(7)
Co6^{viii}—Mn2—Co1^{i}	142.672(14)	Ge1—Co3—Co4^{iii}	179.127(14)
Co3^{v}—Mn2—Co1^{i}	119.124(16)	Ge2^{vii}—Co3—Co4^{iii}	58.850(12)
Co3^{iv}—Mn2—Co1^{i}	96.540(12)	Ge3^{xii}—Co3—Co4^{iii}	58.587(10)
Mn1—Mn2—Co1^{ii}	56.251(12)	Co3^{ix}—Co3—Co3^{xvii}	180.000
Ge2^{viii}—Mn2—Co1^{ii}	147.504(13)	Ge1—Co3—Co3^{xvii}	119.564(7)
Co6^{viii}—Mn2—Co1^{ii}	142.672(14)	Ge2^{vii}—Co3—Co3^{xvii}	58.331(7)
Co3^{v}—Mn2—Co1^{ii}	96.540(12)	Ge3^{xii}—Co3—Co3^{xvii}	58.385(6)
Co3^{iv}—Mn2—Co1^{ii}	119.124(16)	Co4^{iii}—Co3—Co3^{xvii}	59.893(7)
Co1^{i}—Mn2—Co1^{ii}	48.370(13)	Co3^{ix}—Co3—Mn6	65.100(7)
Mn1—Mn2—Ge1^{iv}	98.916(12)	Ge1—Co3—Mn6	117.135(12)
Ge2^{viii}—Mn2—Ge1^{iv}	96.218(11)	Ge2^{vii}—Co3—Mn6	113.515(14)
Co6^{viii}—Mn2—Ge1^{iv}	117.169(10)	Ge3^{xii}—Co3—Mn6	66.147(10)
Co3^{v}—Mn2—Ge1^{iv}	98.151(14)	Co4^{iii}—Co3—Mn6	63.697(11)
Co3^{iv}—Mn2—Ge1^{iv}	50.046(9)	Co3^{xvii}—Co3—Mn6	114.899(7)
Co1^{i}—Mn2—Ge1^{iv}	51.924(9)	Co3^{ix}—Co3—Mn2^{xix}	117.066(7)
Co1^{ii}—Mn2—Ge1^{iv}	95.115(15)	Ge1—Co3—Mn2^{xix}	66.001(11)

Mn1—Mn2—Ge1^{iii}	98.916(12)	Ge2^{vii}—Co3—Mn2^{xix}	63.373(12)
Ge2^{viii}—Mn2—Ge1^{iii}	96.218(11)	Ge3^{xii}—Co3—Mn2^{xix}	113.298(12)
Co6^{viii}—Mn2—Ge1^{iii}	117.169(10)	Co4^{iii}—Co3—Mn2^{xix}	113.163(13)
Co3^{v}—Mn2—Ge1^{iii}	50.046(9)	Co3^{xvii}—Co3—Mn2^{xix}	62.935(7)
Co3^{iv}—Mn2—Ge1^{iii}	98.151(14)	Mn6—Co3—Mn2^{xix}	176.744(13)
Co1^{i}—Mn2—Ge1^{iii}	95.115(15)	Co3^{ix}—Co3—Mn6^{viii}	65.305(6)
Co1^{ii}—Mn2—Ge1^{iii}	51.924(9)	Ge1—Co3—Mn6^{viii}	66.354(13)
Ge1^{iv}—Mn2—Ge1^{iii}	118.966(19)	Ge2^{vii}—Co3—Mn6^{viii}	171.704(12)
Mn1—Mn2—M2^{iv}	57.446(13)	Ge3^{xii}—Co3—Mn6^{viii}	65.785(10)
Ge2^{viii}—Mn2—M2^{iv}	95.632(15)	Co4^{iii}—Co3—Mn6^{viii}	114.451(15)
Co6^{viii}—Mn2—M2^{iv}	49.533(10)	Co3^{xvii}—Co3—Mn6^{viii}	114.696(7)
Co3^{v}—Mn2—M2^{iv}	145.929(17)	Mn6—Co3—Mn6^{viii}	64.120(15)
Co3^{iv}—Mn2—M2^{iv}	116.717(12)	Mn2^{xix}—Co3—Mn6^{viii}	118.807(14)
Co1^{i}—Mn2—M2^{iv}	93.490(13)	Co3^{ix}—Co3—Mn5	65.531(6)
Co1^{ii}—Mn2—M2^{iv}	113.697(16)	Ge1—Co3—Mn5	65.811(13)
Ge1^{iv}—Mn2—M2^{iv}	94.547(10)	Ge2^{vii}—Co3—Mn5	65.067(10)
Ge1^{iii}—Mn2—M2^{iv}	142.787(13)	Ge3^{xii}—Co3—Mn5	170.964(12)
Mn1—Mn2—M2^{v}	57.446(13)	Co4^{iii}—Co3—Mn5	113.676(16)
Ge2^{viii}—Mn2—M2^{v}	95.632(16)	Co3^{xvii}—Co3—Mn5	114.469(6)
Co6^{viii}—Mn2—M2^{v}	49.533(10)	Mn6—Co3—Mn5	115.895(13)
Co3^{v}—Mn2—M2^{v}	116.717(12)	Mn2^{xix}—Co3—Mn5	64.117(11)
Co3^{iv}—Mn2—M2^{v}	145.929(17)	Mn6^{viii}—Co3—Mn5	123.228(12)
Co1^{i}—Mn2—M2^{v}	113.697(16)	Co3^{ix}—Co3—Mn4	65.621(6)
Co1^{ii}—Mn2—M2^{v}	93.490(13)	Ge1—Co3—Mn4	116.495(12)
Ge1^{iv}—Mn2—M2^{v}	142.787(13)	Ge2^{vii}—Co3—Mn4	65.622(11)
Ge1^{iii}—Mn2—M2^{v}	94.547(10)	Ge3^{xii}—Co3—Mn4	113.493(12)
M2^{iv}—Mn2—M2^{v}	49.192(12)	Co4^{iii}—Co3—Mn4	63.555(11)
Mn1—Mn2—Mn3^{i}	108.343(19)	Co3^{xvii}—Co3—Mn4	114.378(6)
Ge2^{viii}—Mn2—Mn3^{i}	101.744(18)	Mn6—Co3—Mn4	60.763(12)
Co6^{viii}—Mn2—Mn3^{i}	153.483(19)	Mn2^{xix}—Co3—Mn4	117.449(14)
Co3^{v}—Mn2—Mn3^{i}	59.994(12)	Mn6^{viii}—Co3—Mn4	116.988(13)
Co3^{iv}—Mn2—Mn3^{i}	59.994(12)	Mn5—Co3—Mn4	63.085(14)
Co1^{i}—Mn2—Mn3^{i}	59.207(13)	Co3^{ix}—Co3—Mn3^{xii}	116.428(6)
Co1^{ii}—Mn2—Mn3^{i}	59.207(13)	Ge1—Co3—Mn3^{xii}	65.340(11)

$Ge1^{iv}$—Mn2—$Mn3^{i}$	59.485(9)	$Ge2^{vii}$—Co3—$Mn3^{xii}$	112.779(12)
$Ge1^{iii}$—Mn2—$Mn3^{i}$	59.485(9)	$Ge3^{xii}$—Co3—$Mn3^{xii}$	65.429(11)
$M2^{iv}$—Mn2—$Mn3^{i}$	149.889(11)	$Co4^{iii}$—Co3—$Mn3^{xii}$	114.595(12)
$M2^{v}$—Mn2—$Mn3^{i}$	149.889(11)	$Co3^{xvii}$—Co3—$Mn3^{xii}$	63.573(6)
Co4—Mn3—$Co1^{ix}$	146.871(13)	Mn6—Co3—$Mn3^{xii}$	119.562(14)
Co4—Mn3—Co1	146.871(13)	$Mn2^{xix}$—Co3—$Mn3^{xii}$	62.115(12)
$Co1^{ix}$—Mn3—Co1	47.944(13)	$Mn6^{viii}$—Co3—$Mn3^{xii}$	64.396(12)
Co4—Mn3—$Co6^{vii}$	93.926(12)	Mn5—Co3—$Mn3^{xii}$	117.693(14)
$Co1^{ix}$—Mn3—$Co6^{vii}$	96.253(15)	Mn4—Co3—$Mn3^{xii}$	177.938(10)
Co1—Mn3—$Co6^{vii}$	53.755(10)	$M2^{ix}$—Co4—M2	62.515(14)
Co4—Mn3—$Co6^{vi}$	93.926(12)	$M2^{ix}$—Co4—Ge3	122.390(13)
$Co1^{ix}$—Mn3—$Co6^{vi}$	53.755(10)	M2—Co4—Ge3	122.390(13)
Co1—Mn3—$Co6^{vi}$	96.252(15)	$M2^{ix}$—Co4—$Ge2^{xiv}$	120.047(15)
$Co6^{vii}$—Mn3—$Co6^{vi}$	119.00(2)	M2—Co4—$Ge2^{xiv}$	120.047(15)
Co4—Mn3—Mn5	101.011(17)	Ge3—Co4—$Ge2^{xiv}$	105.342(14)
$Co1^{ix}$—Mn3—Mn5	58.751(12)	$M2^{ix}$—Co4—$Co3^{xviii}$	118.617(9)
Co1—Mn3—Mn5	58.750(12)	M2—Co4—$Co3^{xviii}$	178.209(15)
$Co6^{vii}$—Mn3—Mn5	59.556(10)	Ge3—Co4—$Co3^{xviii}$	58.458(10)
$Co6^{vi}$—Mn3—Mn5	59.556(10)	$Ge2^{xiv}$—Co4—$Co3^{xviii}$	58.262(11)
Co4—Mn3—$Co3^{x}$	94.438(14)	$M2^{ix}$—Co4—$Co3^{xx}$	178.209(15)
$Co1^{ix}$—Mn3—$Co3^{x}$	94.616(12)	M2—Co4—$Co3^{xx}$	118.618(10)
Co1—Mn3—$Co3^{x}$	116.241(15)	Ge3—Co4—$Co3^{xx}$	58.458(10)
$Co6^{vii}$—Mn3—$Co3^{x}$	145.774(13)	$Ge2^{xiv}$—Co4—$Co3^{xx}$	58.262(11)
$Co6^{vi}$—Mn3—$Co3^{x}$	93.431(11)	$Co3^{xviii}$—Co4—$Co3^{xx}$	60.213(14)
Mn5—Mn3—$Co3^{x}$	149.423(11)	$M2^{ix}$—Co4—$Mn7^{xiv}$	65.692(13)
Co4—Mn3—$Co3^{vii}$	94.438(14)	M2—Co4—$Mn7^{xiv}$	65.691(12)
$Co1^{ix}$—Mn3—$Co3^{vii}$	116.240(15)	Ge3—Co4—$Mn7^{xiv}$	169.984(17)
Co1—Mn3—$Co3^{vii}$	94.617(12)	$Ge2^{xiv}$—Co4—$Mn7^{xiv}$	64.642(13)
$Co6^{vii}$—Mn3—$Co3^{vii}$	93.431(11)	$Co3^{xviii}$—Co4—$Mn7^{xiv}$	113.286(15)
$Co6^{vi}$—Mn3—$Co3^{vii}$	145.774(13)	$Co3^{xx}$—Co4—$Mn7^{xiv}$	113.286(15)
Mn5—Mn3—$Co3^{vii}$	149.423(11)	$M2^{ix}$—Co4—Mn3	66.490(13)
$Co3^{x}$—Mn3—$Co3^{vii}$	52.855(13)	M2—Co4—Mn3	66.490(13)
Co4—Mn3—$Ge1^{vi}$	116.264(10)	Ge3—Co4—Mn3	66.620(12)
$Co1^{ix}$—Mn3—$Ge1^{vi}$	51.544(9)	$Ge2^{xiv}$—Co4—Mn3	171.962(18)

Co1—Mn3—Ge1^{vi}	94.261(14)	Co3^{xviii}—Co4—Mn3	115.140(15)
Co6^{vii}—Mn3—Ge1^{vi}	147.255(18)	Co3^{xx}—Co4—Mn3	115.140(15)
Co6^{vi}—Mn3—Ge1^{vi}	50.549(11)	Mn7^{xiv}—Co4—Mn3	123.397(17)
Mn5—Mn3—Ge1^{vi}	100.099(12)	M2^{ix}—Co4—Mn6^{xviii}	65.517(11)
Co3^{x}—Mn3—Ge1^{vi}	49.326(9)	M2—Co4—Mn6^{xviii}	119.095(16)
Co3^{vii}—Mn3—Ge1^{vi}	96.367(14)	Ge3—Co4—Mn6^{xviii}	65.665(10)
Co4—Mn3—Ge1^{vii}	116.264(10)	Ge2^{xiv}—Co4—Mn6^{xviii}	112.122(11)
Co1^{ix}—Mn3—Ge1^{vii}	94.261(14)	Co3^{xviii}—Co4—Mn6^{xviii}	62.629(12)
Co1—Mn3—Ge1^{vii}	51.545(9)	Co3^{xx}—Co4—Mn6^{xviii}	114.263(16)
Co6^{vii}—Mn3—Ge1^{vii}	50.549(11)	Mn7^{xiv}—Co4—Mn6^{xviii}	117.004(10)
Co6^{vi}—Mn3—Ge1^{vii}	147.255(18)	Mn3—Co4—Mn6^{xviii}	65.28(1)
Mn5—Mn3—Ge1^{vii}	100.099(12)	M2^{ix}—Co4—Mn6^{xix}	119.095(16)
Co3^{x}—Mn3—Ge1^{vii}	96.367(15)	M2—Co4—Mn6^{xix}	65.518(11)
Co3^{vii}—Mn3—Ge1^{vii}	49.326(9)	Ge3—Co4—Mn6^{xix}	65.665(10)
Ge1^{vi}—Mn3—Ge1^{vii}	117.917(18)	Ge2^{xiv}—Co4—Mn6^{xix}	112.122(11)
Co4—Mn3—M2^{ix}	50.212(10)	Co3^{xviii}—Co4—Mn6^{xix}	114.263(15)
Co1^{ix}—Mn3—M2^{ix}	97.335(12)	Co3^{xx}—Co4—Mn6^{xix}	62.629(12)
Co1—Mn3—M2^{ix}	118.750(15)	Mn7^{xiv}—Co4—Mn6^{xix}	117.004(10)
Co6^{vii}—Mn3—M2^{ix}	95.732(15)	Mn3—Co4—Mn6^{xix}	65.28(1)
Co6^{vi}—Mn3—M2^{ix}	49.500(9)	Mn6^{xviii}—Co4—Mn6^{xix}	120.427(19)
Mn5—Mn3—M2^{ix}	60.022(12)	M2^{ix}—Co4—Mn4^{xix}	116.542(16)
Co3^{x}—Mn3—M2^{ix}	114.912(12)	M2—Co4—Mn4^{xix}	63.576(12)
Co3^{vii}—Mn3—M2^{ix}	143.927(17)	Ge3—Co4—Mn4^{xix}	113.441(11)
Ge1^{vi}—Mn3—M2^{ix}	94.494(9)	Ge2^{xiv}—Co4—Mn4^{xix}	65.478(10)
Ge1^{vii}—Mn3—M2^{ix}	145.433(13)	Co3^{xviii}—Co4—Mn4^{xix}	114.698(16)
Co4—Mn3—M2	50.213(10)	Co3^{xx}—Co4—Mn4^{xix}	63.620(12)
Co1^{ix}—Mn3—M2	118.750(15)	Mn7^{xiv}—Co4—Mn4^{xix}	63.409(11)
Co1—Mn3—M2	97.334(12)	Mn3—Co4—Mn4^{xix}	116.919(10)
Co6^{vii}—Mn3—M2	49.500(9)	Mn6^{xviii}—Co4—Mn4^{xix}	177.315(17)
Co6^{vi}—Mn3—M2	95.732(15)	Mn6^{xix}—Co4—Mn4^{xix}	60.475(12)
Mn5—Mn3—M2	60.021(12)	M2—Co5—M2^{ix}	62.242(15)
Co3^{x}—Mn3—M2	143.928(17)	M2—Co5—Co1^{xii}	117.043(10)
Co3^{vii}—Mn3—M2	114.913(12)	M2^{ix}—Co5—Co1^{xii}	178.316(18)
Ge1^{vi}—Mn3—M2	145.433(13)	M2—Co5—Co1^{xv}	178.316(18)

Ge1^{vii}—Mn3—M2	94.495(9)	M2^{ix}—Co5—Co1^{xv}	117.043(10)
M2^{ix}—Mn3—M2	51.548(13)	Co1^{xii}—Co5—Co1^{xv}	63.625(15)
Co1—Mn4—Co1^{ix}	50.280(14)	M2—Co5—Ge1	118.946(14)
Co1—Mn4—M2^{v}	115.231(13)	M2^{ix}—Co5—Ge1	118.946(14)
Co1^{ix}—Mn4—M2^{v}	142.520(19)	Co1^{xii}—Co5—Ge1	59.854(11)
Co1—Mn4—M2^{iv}	142.521(19)	Co1^{xv}—Co5—Ge1	59.854(11)
Co1^{ix}—Mn4—M2^{iv}	115.231(13)	M2—Co5—Mn7	117.980(16)
M2^{v}—Mn4—M2^{iv}	51.226(13)	M2^{ix}—Co5—Mn7	117.980(15)
Co1—Mn4—Co4^{iii}	94.947(10)	Co1^{xii}—Co5—Mn7	63.695(13)
Co1^{ix}—Mn4—Co4^{iii}	144.729(13)	Co1^{xv}—Co5—Mn7	63.695(13)
M2^{v}—Mn4—Co4^{iii}	50.256(9)	Ge1—Co5—Mn7	112.341(18)
M2^{iv}—Mn4—Co4^{iii}	95.949(15)	M2—Co5—Mn7^{xiv}	66.610(12)
Co1—Mn4—Co4^{iv}	144.729(13)	M2^{ix}—Co5—Mn7^{xiv}	66.610(12)
Co1^{ix}—Mn4—Co4^{iv}	94.948(10)	Co1^{xii}—Co5—Mn7^{xiv}	114.678(15)
M2^{v}—Mn4—Co4^{iv}	95.949(15)	Co1^{xv}—Co5—Mn7^{xiv}	114.678(15)
M2^{iv}—Mn4—Co4^{iv}	50.256(9)	Ge1—Co5—Mn7^{xiv}	173.201(19)
Co4^{iii}—Mn4—Co4^{iv}	118.49(2)	Mn7—Co5—Mn7^{xiv}	60.861(17)
Co1—Mn4—Co3^{ix}	116.840(16)	M2—Co5—Mn1^{xviii}	119.820(16)
Co1^{ix}—Mn4—Co3^{ix}	95.785(13)	M2^{ix}—Co5—Mn1^{xviii}	63.598(12)
M2^{v}—Mn4—Co3^{ix}	119.162(16)	Co1^{xii}—Co5—Mn1^{xviii}	117.724(17)
M2^{iv}—Mn4—Co3^{ix}	97.493(12)	Co1^{xv}—Co5—Mn1^{xviii}	60.298(12)
Co4^{iii}—Mn4—Co3^{ix}	96.038(15)	Ge1—Co5—Mn1^{xviii}	108.748(12)
Co4^{iv}—Mn4—Co3^{ix}	52.824(10)	Mn7—Co5—Mn1^{xviii}	68.876(11)
Co1—Mn4—Co3	95.785(13)	Mn7^{xiv}—Co5—Mn1^{xviii}	69.535(11)
Co1^{ix}—Mn4—Co3	116.840(16)	M2—Co5—Mn1^{xix}	63.598(12)
M2^{v}—Mn4—Co3	97.494(12)	M2^{ix}—Co5—Mn1^{xix}	119.820(16)
M2^{iv}—Mn4—Co3	119.162(16)	Co1^{xii}—Co5—Mn1^{xix}	60.298(12)
Co4^{iii}—Mn4—Co3	52.825(10)	Co1^{xv}—Co5—Mn1^{xix}	117.724(17)
Co4^{iv}—Mn4—Co3	96.038(15)	Ge1—Co5—Mn1^{xix}	108.748(12)
Co3^{ix}—Mn4—Co3	48.757(13)	Mn7—Co5—Mn1^{xix}	68.876(11)
Co1—Mn4—Mn6	150.847(11)	Mn7^{xiv}—Co5—Mn1^{xix}	69.535(12)
Co1^{ix}—Mn4—Mn6	150.847(11)	Mn1^{xviii}—Co5—Mn1^{xix}	131.36(2)
M2^{v}—Mn4—Mn6	60.518(12)	M2—Co5—Mn5	64.902(13)
M2^{iv}—Mn4—Mn6	60.518(12)	M2^{ix}—Co5—Mn5	64.902(13)

$Co4^{iii}$—Mn4—Mn6	59.278(10)	$Co1^{xii}$—Co5—Mn5	113.431(15)
$Co4^{iv}$—Mn4—Mn6	59.278(10)	$Co1^{xv}$—Co5—Mn5	113.431(15)
$Co3^{ix}$—Mn4—Mn6	58.649(13)	Ge1—Co5—Mn5	64.128(15)
Co3—Mn4—Mn6	58.649(13)	Mn7—Co5—Mn5	176.469(18)
Co1—Mn4—$Ge2^{vi}$	95.845(14)	$Mn7^{xiv}$—Co5—Mn5	122.671(18)
$Co1^{ix}$—Mn4—$Ge2^{vi}$	51.646(9)	$Mn1^{xviii}$—Co5—Mn5	111.838(11)
$M2^{v}$—Mn4—$Ge2^{vi}$	145.618(12)	$Mn1^{xix}$—Co5—Mn5	111.838(11)
$M2^{iv}$—Mn4—$Ge2^{vi}$	95.178(10)	M2—Co5—$Mn2^{xix}$	65.075(11)
$Co4^{iii}$—Mn4—$Ge2^{vi}$	145.415(19)	$M2^{ix}$—Co5—$Mn2^{xix}$	116.430(16)
$Co4^{iv}$—Mn4—$Ge2^{vi}$	50.361(10)	$Co1^{xii}$—Co5—$Mn2^{xix}$	62.083(11)
$Co3^{ix}$—Mn4—$Ge2^{vi}$	50.010(9)	$Co1^{xv}$—Co5—$Mn2^{xix}$	114.558(15)
Co3—Mn4—$Ge2^{vi}$	93.393(14)	Ge1—Co5—$Mn2^{xix}$	62.781(10)
Mn6—Mn4—$Ge2^{vi}$	99.209(12)	Mn7—Co5—$Mn2^{xix}$	116.636(11)
Co1—Mn4—$Ge2^{vii}$	51.646(9)	$Mn7^{xiv}$—Co5—$Mn2^{xix}$	119.295(10)
$Co1^{ix}$—Mn4—$Ge2^{vii}$	95.845(14)	$Mn1^{xviii}$—Co5—$Mn2^{xix}$	170.869(17)
$M2^{v}$—Mn4—$Ge2^{vii}$	95.178(10)	$Mn1^{xix}$—Co5—$Mn2^{xix}$	57.285(12)
$M2^{iv}$—Mn4—$Ge2^{vii}$	145.618(12)	Mn5—Co5—$Mn2^{xix}$	62.188(10)
$Co4^{iii}$—Mn4—$Ge2^{vii}$	50.361(10)	$M2^{xii}$—Co6—$M2^{xv}$	60.705(15)
$Co4^{iv}$—Mn4—$Ge2^{vii}$	145.415(19)	$M2^{xii}$—Co6—Ge2	121.286(15)
$Co3^{ix}$—Mn4—$Ge2^{vii}$	93.393(14)	$M2^{xv}$—Co6—Ge2	121.286(15)
Co3—Mn4—$Ge2^{vii}$	50.010(9)	$M2^{xii}$—Co6—Ge1	121.102(15)
Mn6—Mn4—$Ge2^{vii}$	99.209(12)	$M2^{xv}$—Co6—Ge1	121.102(15)
$Ge2^{vi}$—Mn4—$Ge2^{vii}$	116.460(18)	Ge2—Co6—Ge1	106.231(16)
Co1—Mn4—$Mn7^{vi}$	99.475(16)	$M2^{xii}$—Co6—$Co1^{xv}$	179.656(18)
$Co1^{ix}$—Mn4—$Mn7^{vi}$	56.999(12)	$M2^{xv}$—Co6—$Co1^{xv}$	119.185(10)
$M2^{v}$—Mn4—$Mn7^{vi}$	101.374(15)	Ge2—Co6—$Co1^{xv}$	58.449(11)
$M2^{iv}$—Mn4—$Mn7^{vi}$	58.365(11)	Ge1—Co6—$Co1^{xv}$	59.242(11)
$Co4^{iii}$—Mn4—$Mn7^{vi}$	151.623(19)	$M2^{xii}$—Co6—$Co1^{xii}$	119.185(10)
$Co4^{iv}$—Mn4—$Mn7^{vi}$	56.293(11)	$M2^{xv}$—Co6—$Co1^{xii}$	179.656(18)
$Co3^{ix}$—Mn4—$Mn7^{vi}$	98.993(11)	Ge2—Co6—$Co1^{xii}$	58.449(11)
Co3—Mn4—$Mn7^{vi}$	147.720(13)	Ge1—Co6—$Co1^{xii}$	59.242(11)
Mn6—Mn4—$Mn7^{vi}$	109.666(13)	$Co1^{xv}$—Co6—$Co1^{xii}$	60.924(15)
$Ge2^{vi}$—Mn4—$Mn7^{vi}$	56.978(10)	$M2^{xii}$—Co6—$Mn2^{viii}$	67.463(13)
$Ge2^{vii}$—Mn4—$Mn7^{vi}$	150.976(18)	$M2^{xv}$—Co6—$Mn2^{viii}$	67.463(13)

Co1—Mn5—Co1^{ix}	48.663(13)	Ge2—Co6—Mn2^{viii}	63.368(13)
Co1—Mn5—Co6^{vi}	97.414(15)	Ge1—Co6—Mn2^{viii}	169.598(18)
Co1^{ix}—Mn5—Co6^{vi}	54.276(10)	Co1^{xv}—Co6—Mn2^{viii}	112.193(14)
Co1—Mn5—Co6^{vii}	54.276(10)	Co1^{xii}—Co6—Mn2^{viii}	112.193(14)
Co1^{ix}—Mn5—Co6^{vii}	97.413(15)	M2^{xii}—Co6—Mn5^{xii}	65.815(12)
Co6^{vi}—Mn5—Co6^{vii}	119.80(2)	M2^{xv}—Co6—Mn5^{xii}	117.688(16)
Co1—Mn5—Co3^{ix}	114.443(16)	Ge2—Co6—Mn5^{xii}	65.234(10)
Co1^{ix}—Mn5—Co3^{ix}	94.156(13)	Ge1—Co6—Mn5^{xii}	112.450(11)
Co6^{vi}—Mn5—Co3^{ix}	94.978(10)	Co1^{xv}—Co6—Mn5^{xii}	114.098(16)
Co6^{vii}—Mn5—Co3^{ix}	143.463(13)	Co1^{xii}—Co6—Mn5^{xii}	62.037(11)
Co1—Mn5—Co3	94.156(13)	Mn2^{viii}—Co6—Mn5^{xii}	64.259(10)
Co1^{ix}—Mn5—Co3	114.444(16)	M2^{xii}—Co6—Mn5^{xiii}	117.688(16)
Co6^{vi}—Mn5—Co3	143.463(13)	M2^{xv}—Co6—Mn5^{xiii}	65.815(11)
Co6^{vii}—Mn5—Co3	94.979(10)	Ge2—Co6—Mn5^{xiii}	65.234(10)
Co3^{ix}—Mn5—Co3	48.938(13)	Ge1—Co6—Mn5^{xiii}	112.450(11)
Co1—Mn5—Ge2^{vii}	51.190(9)	Co1^{xv}—Co6—Mn5^{xiii}	62.037(12)
Co1^{ix}—Mn5—Ge2^{vii}	94.622(14)	Co1^{xii}—Co6—Mn5^{xiii}	114.098(16)
Co6^{vi}—Mn5—Ge2^{vii}	148.097(18)	Mn2^{viii}—Co6—Mn5^{xiii}	64.259(10)
Co6^{vii}—Mn5—Ge2^{vii}	50.484(10)	Mn5^{xii}—Co6—Mn5^{xiii}	119.80(2)
Co3^{ix}—Mn5—Ge2^{vii}	94.195(14)	M2^{xii}—Co6—Mn6^{viii}	65.454(13)
Co3—Mn5—Ge2^{vii}	50.364(9)	M2^{xv}—Co6—Mn6^{viii}	65.454(13)
Co1—Mn5—Ge2^{vi}	94.622(14)	Ge2—Co6—Mn6^{viii}	171.779(18)
Co1^{ix}—Mn5—Ge2^{vi}	51.190(9)	Ge1—Co6—Mn6^{viii}	65.548(14)
Co6^{vi}—Mn5—Ge2^{vi}	50.484(10)	Co1^{xv}—Co6—Mn6^{viii}	114.831(15)
Co6^{vii}—Mn5—Ge2^{vi}	148.097(18)	Co1^{xii}—Co6—Mn6^{viii}	114.831(15)
Co3^{ix}—Mn5—Ge2^{vi}	50.363(9)	Mn2^{viii}—Co6—Mn6^{viii}	124.853(17)
Co3—Mn5—Ge2^{vi}	94.195(14)	Mn5^{xii}—Co6—Mn6^{viii}	117.003(10)
Ge2^{vii}—Mn5—Ge2^{vi}	118.267(18)	Mn5^{xiii}—Co6—Mn6^{viii}	117.003(10)
Co1—Mn5—Mn3	60.110(13)	M2^{xii}—Co6—Mn3^{xiii}	117.246(16)
Co1^{ix}—Mn5—Mn3	60.109(13)	M2^{xv}—Co6—Mn3^{xiii}	65.606(12)
Co6^{vi}—Mn5—Mn3	59.955(10)	Ge2—Co6—Mn3^{xiii}	112.902(11)
Co6^{vii}—Mn5—Mn3	59.955(10)	Ge1—Co6—Mn3^{xiii}	65.069(10)
Co3^{ix}—Mn5—Mn3	150.980(11)	Co1^{xv}—Co6—Mn3^{xiii}	62.850(11)
Co3—Mn5—Mn3	150.980(11)	Co1^{xii}—Co6—Mn3^{xiii}	114.667(16)

Ge2^{vii}—Mn5—Mn3	100.643(12)	Mn2^{viii}—Co6—Mn3^{xiii}	117.750(11)
Ge2^{vi}—Mn5—Mn3	100.643(12)	Mn5^{xii}—Co6—Mn3^{xiii}	176.662(18)
Co1—Mn5—M2	98.232(12)	Mn5^{xiii}—Co6—Mn3^{xiii}	60.488(11)
Co1^{ix}—Mn5—M2	120.095(16)	Mn6^{viii}—Co6—Mn3^{xiii}	64.422(11)
Co6^{vi}—Mn5—M2	95.980(14)	M2^{xii}—Co6—Mn3^{xii}	65.606(12)
Co6^{vii}—Mn5—M2	49.602(9)	M2^{xv}—Co6—Mn3^{xii}	117.246(16)
Co3^{ix}—Mn5—M2	143.742(17)	Ge2—Co6—Mn3^{xii}	112.902(11)
Co3—Mn5—M2	116.500(12)	Ge1—Co6—Mn3^{xii}	65.069(11)
Ge2^{vii}—Mn5—M2	94.455(9)	Co1^{xv}—Co6—Mn3^{xii}	114.667(16)
Ge2^{vi}—Mn5—M2	145.492(12)	Co1^{xii}—Co6—Mn3^{xii}	62.850(12)
Mn3—Mn5—M2	60.009(12)	Mn2^{viii}—Co6—Mn3^{xii}	117.75(1)
Co1—Mn5—M2^{ix}	120.095(16)	Mn5^{xii}—Co6—Mn3^{xii}	60.488(11)
Co1^{ix}—Mn5—M2^{ix}	98.231(12)	Mn5^{xiii}—Co6—Mn3^{xii}	176.662(18)
Co6^{vi}—Mn5—M2^{ix}	49.602(9)	Mn6^{viii}—Co6—Mn3^{xii}	64.422(11)
Co6^{vii}—Mn5—M2^{ix}	95.980(15)	Mn3^{xiii}—Co6—Mn3^{xii}	119.01(2)
Co3^{ix}—Mn5—M2^{ix}	116.500(12)	Co3—Ge1—Co3^{ix}	59.126(14)
Co3—Mn5—M2^{ix}	143.742(17)	Co3—Ge1—Co6	118.866(14)
Ge2^{vii}—Mn5—M2^{ix}	145.492(12)	Co3^{ix}—Ge1—Co6	118.866(14)
Ge2^{vi}—Mn5—M2^{ix}	94.455(10)	Co3—Ge1—Co1^{xv}	177.514(12)
Mn3—Mn5—M2^{ix}	60.008(12)	Co3^{ix}—Ge1—Co1^{xv}	118.71(1)
M2—Mn5—M2^{ix}	51.540(12)	Co6—Ge1—Co1^{xv}	62.997(11)
Co3^{ix}—Mn6—Co3	49.800(13)	Co3—Ge1—Co1^{xii}	118.71(1)
Co3^{ix}—Mn6—Co3^{xi}	115.880(15)	Co3^{ix}—Ge1—Co1^{xii}	177.514(12)
Co3—Mn6—Co3^{xi}	94.856(13)	Co6—Ge1—Co1^{xii}	62.997(11)
Co3^{ix}—Mn6—Co3^{viii}	94.857(13)	Co1^{xv}—Ge1—Co1^{xii}	63.422(14)
Co3—Mn6—Co3^{viii}	115.879(15)	Co3—Ge1—Co5	119.993(14)
Co3^{xi}—Mn6—Co3^{viii}	49.392(13)	Co3^{ix}—Ge1—Co5	119.992(14)
Co3^{ix}—Mn6—Co4^{iv}	53.674(10)	Co6—Ge1—Co5	111.209(16)
Co3—Mn6—Co4^{iv}	97.957(15)	Co1^{xv}—Ge1—Co5	59.574(11)
Co3^{xi}—Mn6—Co4^{iv}	143.550(13)	Co1^{xii}—Ge1—Co5	59.574(11)
Co3^{viii}—Mn6—Co4^{iv}	94.541(10)	Co3—Ge1—Mn2^{xviii}	114.265(15)
Co3^{ix}—Mn6—Co4^{iii}	97.957(15)	Co3^{ix}—Ge1—Mn2^{xviii}	63.952(11)
Co3—Mn6—Co4^{iii}	53.674(10)	Co6—Ge1—Mn2^{xviii}	115.542(10)
Co3^{xi}—Mn6—Co4^{iii}	94.541(11)	Co1^{xv}—Ge1—Mn2^{xviii}	63.255(11)

Co3^{viii}—Mn6—Co4^{iii}	143.550(13)	Co1^{xii}—Ge1—Mn2^{xviii}	117.105(14)
Co4^{iv}—Mn6—Co4^{iii}	120.43(2)	Co5—Ge1—Mn2^{xviii}	66.043(10)
Co3^{ix}—Mn6—Co6^{viii}	146.315(13)	Co3—Ge1—Mn2^{xix}	63.952(11)
Co3—Mn6—Co6^{viii}	146.316(13)	Co3^{ix}—Ge1—Mn2^{xix}	114.265(14)
Co3^{xi}—Mn6—Co6^{viii}	94.561(15)	Co6—Ge1—Mn2^{xix}	115.542(10)
Co3^{viii}—Mn6—Co6^{viii}	94.561(15)	Co1^{xv}—Ge1—Mn2^{xix}	117.105(14)
Co4^{iv}—Mn6—Co6^{viii}	93.369(12)	Co1^{xii}—Ge1—Mn2^{xix}	63.255(11)
Co4^{iii}—Mn6—Co6^{viii}	93.369(12)	Co5—Ge1—Mn2^{xix}	66.043(10)
Co3^{ix}—Mn6—M2^{iv}	97.499(12)	Mn2^{xviii}—Ge1—Mn2^{xix}	118.966(19)
Co3—Mn6—M2^{iv}	119.169(16)	Co3—Ge1—Mn3^{xii}	65.334(12)
Co3^{xi}—Mn6—M2^{iv}	143.380(17)	Co3^{ix}—Ge1—Mn3^{xii}	115.349(14)
Co3^{viii}—Mn6—M2^{iv}	116.646(12)	Co6—Ge1—Mn3^{xii}	64.382(10)
Co4^{iv}—Mn6—M2^{iv}	50.003(9)	Co1^{xv}—Ge1—Mn3^{xii}	117.143(14)
Co4^{iii}—Mn6—M2^{iv}	95.330(15)	Co1^{xii}—Ge1—Mn3^{xii}	63.608(12)
Co6^{viii}—Mn6—M2^{iv}	49.721(10)	Co5—Ge1—Mn3^{xii}	114.733(10)
Co3^{ix}—Mn6—M2^{v}	119.168(15)	Mn2^{xviii}—Ge1—Mn3^{xii}	179.217(15)
Co3—Mn6—M2^{v}	97.499(12)	Mn2^{xix}—Ge1—Mn3^{xii}	61.555(11)
Co3^{xi}—Mn6—M2^{v}	116.646(12)	Co3—Ge1—Mn3^{xiii}	115.349(14)
Co3^{viii}—Mn6—M2^{v}	143.380(17)	Co3^{ix}—Ge1—Mn3^{xiii}	65.335(12)
Co4^{iv}—Mn6—M2^{v}	95.330(15)	Co6—Ge1—Mn3^{xiii}	64.382(10)
Co4^{iii}—Mn6—M2^{v}	50.003(9)	Co1^{xv}—Ge1—Mn3^{xiii}	63.608(12)
Co6^{viii}—Mn6—M2^{v}	49.721(10)	Co1^{xii}—Ge1—Mn3^{xiii}	117.143(14)
M2^{iv}—Mn6—M2^{v}	50.150(12)	Co5—Ge1—Mn3^{xiii}	114.733(10)
Co3^{ix}—Mn6—Mn4	60.587(13)	Mn2^{xviii}—Ge1—Mn3^{xiii}	61.555(12)
Co3—Mn6—Mn4	60.588(13)	Mn2^{xix}—Ge1—Mn3^{xiii}	179.217(15)
Co3^{xi}—Mn6—Mn4	151.666(11)	Mn3^{xii}—Ge1—Mn3^{xiii}	117.917(18)
Co3^{viii}—Mn6—Mn4	151.666(11)	Co3—Ge1—Mn5	64.774(12)
Co4^{iv}—Mn6—Mn4	60.246(10)	Co3^{ix}—Ge1—Mn5	64.773(12)
Co4^{iii}—Mn6—Mn4	60.246(10)	Co6—Ge1—Mn5	175.628(16)
Co6^{viii}—Mn6—Mn4	99.327(18)	Co1^{xv}—Ge1—Mn5	113.445(13)
M2^{iv}—Mn6—Mn4	58.587(13)	Co1^{xii}—Ge1—Mn5	113.445(13)
M2^{v}—Mn6—Mn4	58.587(13)	Co5—Ge1—Mn5	64.419(13)
Co3^{ix}—Mn6—Ge3^{xii}	94.968(13)	Mn2^{xviii}—Ge1—Mn5	63.233(10)
Co3—Mn6—Ge3^{xii}	50.739(7)	Mn2^{xix}—Ge1—Mn5	63.233(10)

$Co3^{xi}—Mn6—Ge3^{xii}$	50.543(7)	$Mn3^{xii}—Ge1—Mn5$	116.906(10)
$Co3^{viii}—Mn6—Ge3^{xii}$	94.498(13)	$Mn3^{xiii}—Ge1—Mn5$	116.906(10)
$Co4^{iv}—Mn6—Ge3^{xii}$	147.984(17)	$Co3—Ge1—Mn6^{viii}$	64.053(12)
$Co4^{iii}—Mn6—Ge3^{xii}$	50.573(9)	$Co3^{ix}—Ge1—Mn6^{viii}$	64.054(12)
$Co6^{viii}—Mn6—Ge3^{xii}$	116.411(9)	$Co6—Ge1—Mn6^{viii}$	63.912(13)
$M2^{iv}—Mn6—Ge3^{xii}$	145.095(11)	$Co1^{xv}—Ge1—Mn6^{viii}$	116.517(14)
$M2^{v}—Mn6—Ge3^{xii}$	95.504(8)	$Co1^{xii}—Ge1—Mn6^{viii}$	116.517(14)
$Mn4—Mn6—Ge3^{xii}$	101.131(11)	$Co5—Ge1—Mn6^{viii}$	175.121(16)
$Co3^{ix}—Mn6—Ge3^{xiii}$	50.740(7)	$Mn2^{xviii}—Ge1—Mn6^{viii}$	115.50(1)
$Co3—Mn6—Ge3^{xiii}$	94.968(13)	$Mn2^{xix}—Ge1—Mn6^{viii}$	115.50(1)
$Co3^{xi}—Mn6—Ge3^{xiii}$	94.498(13)	$Mn3^{xii}—Ge1—Mn6^{viii}$	63.736(10)
$Co3^{viii}—Mn6—Ge3^{xiii}$	50.543(7)	$Mn3^{xiii}—Ge1—Mn6^{viii}$	63.736(10)
$Co4^{iv}—Mn6—Ge3^{xiii}$	50.573(9)	$Mn5—Ge1—Mn6^{viii}$	120.460(15)
$Co4^{iii}—Mn6—Ge3^{xiii}$	147.984(17)	$Co3^{xii}—Ge2—Co3^{xv}$	63.336(15)
$Co6^{viii}—Mn6—Ge3^{xiii}$	116.411(9)	$Co3^{xii}—Ge2—Co6$	119.232(13)
$M2^{iv}—Mn6—Ge3^{xiii}$	95.504(8)	$Co3^{xv}—Ge2—Co6$	119.232(13)
$M2^{v}—Mn6—Ge3^{xiii}$	145.095(11)	$Co3^{xii}—Ge2—Co4^{xiv}$	62.888(11)
$Mn4—Mn6—Ge3^{xiii}$	101.131(11)	$Co3^{xv}—Ge2—Co4^{xiv}$	62.888(11)
$Ge3^{xii}—Mn6—Ge3^{xiii}$	117.382(15)	$Co6—Ge2—Co4^{xiv}$	177.361(17)
$Co5—Mn7—Co5^{xiv}$	119.139(17)	$Co3^{xii}—Ge2—Co1^{xv}$	177.093(15)
$Co5—Mn7—Mn7^{xiv}$	60.067(17)	$Co3^{xv}—Ge2—Co1^{xv}$	116.039(11)
$Co5^{xiv}—Mn7—Mn7^{xiv}$	59.072(16)	$Co6—Ge2—Co1^{xv}$	63.644(11)
$Co5—Mn7—Co1^{xii}$	55.005(11)	$Co4^{xiv}—Ge2—Co1^{xv}$	114.225(13)
$Co5^{xiv}—Mn7—Co1^{xii}$	149.093(10)	$Co3^{xii}—Ge2—Co1^{xii}$	116.039(11)
$Mn7^{xiv}—Mn7—Co1^{xii}$	106.741(19)	$Co3^{xv}—Ge2—Co1^{xii}$	177.093(15)
$Co5—Mn7—Co1^{xv}$	55.005(11)	$Co6—Ge2—Co1^{xii}$	63.644(11)
$Co5^{xiv}—Mn7—Co1^{xv}$	149.093(9)	$Co4^{xiv}—Ge2—Co1^{xii}$	114.225(13)
$Mn7^{xiv}—Mn7—Co1^{xv}$	106.741(19)	$Co1^{xv}—Ge2—Co1^{xii}$	64.426(15)
$Co1^{xii}—Mn7—Co1^{xv}$	57.596(14)	$Co3^{xii}—Ge2—Mn2^{viii}$	64.913(12)
$Co5—Mn7—Co4^{xiv}$	148.216(19)	$Co3^{xv}—Ge2—Mn2^{viii}$	64.913(12)
$Co5^{xiv}—Mn7—Co4^{xiv}$	92.644(16)	$Co6—Ge2—Mn2^{viii}$	64.893(15)
$Mn7^{xiv}—Mn7—Co4^{xiv}$	151.72(3)	$Co4^{xiv}—Ge2—Mn2^{viii}$	117.746(17)
$Co1^{xii}—Mn7—Co4^{xiv}$	97.961(14)	$Co1^{xv}—Ge2—Mn2^{viii}$	117.633(13)
$Co1^{xv}—Mn7—Co4^{xiv}$	97.961(14)	$Co1^{xii}—Ge2—Mn2^{viii}$	117.633(13)

Co5—Mn7—Ge2	95.625(17)	$Co3^{xii}$—Ge2—Mn7	115.504(14)
$Co5^{xiv}$—Mn7—Ge2	145.236(18)	$Co3^{xv}$—Ge2—Mn7	115.504(14)
$Mn7^{xiv}$—Mn7—Ge2	155.69(3)	Co6—Ge2—Mn7	114.595(16)
$Co1^{xii}$—Mn7—Ge2	52.900(11)	$Co4^{xiv}$—Ge2—Mn7	62.766(14)
$Co1^{xv}$—Mn7—Ge2	52.900(11)	$Co1^{xv}$—Ge2—Mn7	61.958(12)
$Co4^{xiv}$—Mn7—Ge2	52.592(12)	$Co1^{xii}$—Ge2—Mn7	61.958(12)
Co5—Mn7—$M2^{xiv}$	150.316(10)	$Mn2^{viii}$—Ge2—Mn7	179.488(16)
$Co5^{xiv}$—Mn7—$M2^{xiv}$	52.198(11)	$Co3^{xii}$—Ge2—$Mn5^{xii}$	64.568(11)
$Mn7^{xiv}$—Mn7—$M2^{xiv}$	104.19(2)	$Co3^{xv}$—Ge2—$Mn5^{xii}$	118.154(14)
$Co1^{xii}$—Mn7—$M2^{xiv}$	148.941(17)	Co6—Ge2—$Mn5^{xii}$	64.28(1)
$Co1^{xv}$—Mn7—$M2^{xiv}$	115.339(11)	$Co4^{xiv}$—Ge2—$Mn5^{xii}$	116.494(10)
$Co4^{xiv}$—Mn7—$M2^{xiv}$	51.393(10)	$Co1^{xv}$—Ge2—$Mn5^{xii}$	117.515(14)
Ge2—Mn7—$M2^{xiv}$	97.541(15)	$Co1^{xii}$—Ge2—$Mn5^{xii}$	63.053(11)
Co5—Mn7—$M2^{xvi}$	150.316(10)	$Mn2^{viii}$—Ge2—$Mn5^{xii}$	64.411(10)
$Co5^{xiv}$—Mn7—$M2^{xvi}$	52.198(11)	Mn7—Ge2—$Mn5^{xii}$	115.43(1)
$Mn7^{xiv}$—Mn7—$M2^{xvi}$	104.19(2)	$Co3^{xii}$—Ge2—$Mn5^{xiii}$	118.154(14)
$Co1^{xii}$—Mn7—$M2^{xvi}$	115.339(11)	$Co3^{xv}$—Ge2—$Mn5^{xiii}$	64.568(11)
$Co1^{xv}$—Mn7—$M2^{xvi}$	148.941(17)	Co6—Ge2—$Mn5^{xiii}$	64.28(1)
$Co4^{xiv}$—Mn7—$M2^{xvi}$	51.393(10)	$Co4^{xiv}$—Ge2—$Mn5^{xiii}$	116.494(10)
Ge2—Mn7—$M2^{xvi}$	97.541(15)	$Co1^{xv}$—Ge2—$Mn5^{xiii}$	63.053(11)
$M2^{xiv}$—Mn7—$M2^{xvi}$	52.838(13)	$Co1^{xii}$—Ge2—$Mn5^{xiii}$	117.515(14)
Co5—Mn7—$Mn4^{xii}$	107.142(13)	$Mn2^{viii}$—Ge2—$Mn5^{xiii}$	64.411(10)
$Co5^{xiv}$—Mn7—$Mn4^{xii}$	105.230(13)	Mn7—Ge2—$Mn5^{xiii}$	115.43(1)
$Mn7^{xiv}$—Mn7—$Mn4^{xii}$	123.375(10)	$Mn5^{xii}$—Ge2—$Mn5^{xiii}$	118.268(18)
$Co1^{xii}$—Mn7—$Mn4^{xii}$	57.231(11)	$Co3^{xii}$—Ge2—$Mn4^{xiii}$	117.392(14)
$Co1^{xv}$—Mn7—$Mn4^{xii}$	105.248(16)	$Co3^{xv}$—Ge2—$Mn4^{xiii}$	64.367(12)
$Co4^{xiv}$—Mn7—$Mn4^{xii}$	60.297(11)	Co6—Ge2—$Mn4^{xiii}$	114.946(10)
Ge2—Mn7—$Mn4^{xii}$	60.546(10)	$Co4^{xiv}$—Ge2—$Mn4^{xiii}$	64.162(10)
$M2^{xiv}$—Mn7—$Mn4^{xii}$	102.502(15)	$Co1^{xv}$—Ge2—$Mn4^{xiii}$	60.464(11)
$M2^{xvi}$—Mn7—$Mn4^{xii}$	58.240(11)	$Co1^{xii}$—Ge2—$Mn4^{xiii}$	114.421(15)
Co5—Mn7—$Mn4^{xiii}$	107.142(13)	$Mn2^{viii}$—Ge2—$Mn4^{xiii}$	117.669(10)
$Co5^{xiv}$—Mn7—$Mn4^{xiii}$	105.230(13)	Mn7—Ge2—$Mn4^{xiii}$	62.476(10)
$Mn7^{xiv}$—Mn7—$Mn4^{xiii}$	123.375(10)	$Mn5^{xii}$—Ge2—$Mn4^{xiii}$	177.474(16)
$Co1^{xii}$—Mn7—$Mn4^{xiii}$	105.248(16)	$Mn5^{xiii}$—Ge2—$Mn4^{xiii}$	62.582(12)

$Co1^{xv}—Mn7—Mn4^{xiii}$	57.231(11)	$Co3^{xii}—Ge2—Mn4^{xii}$	64.367(12)
$Co4^{xiv}—Mn7—Mn4^{xiii}$	60.297(11)	$Co3^{xv}—Ge2—Mn4^{xii}$	117.392(14)
$Ge2—Mn7—Mn4^{xiii}$	60.546(10)	$Co6—Ge2—Mn4^{xii}$	114.946(10)
$M2^{xiv}—Mn7—Mn4^{xiii}$	58.240(11)	$Co4^{xiv}—Ge2—Mn4^{xii}$	64.162(10)
$M2^{xvi}—Mn7—Mn4^{xiii}$	102.502(15)	$Co1^{xv}—Ge2—Mn4^{xii}$	114.421(15)
$Mn4^{xii}—Mn7—Mn4^{xiii}$	113.18(2)	$Co1^{xii}—Ge2—Mn4^{xii}$	60.464(11)
$Co5—Mn7—Mn1^{xviii}$	56.307(10)	$Mn2^{viii}—Ge2—Mn4^{xii}$	117.669(10)
$Co5^{xiv}—Mn7—Mn1^{xviii}$	96.591(13)	$Mn7—Ge2—Mn4^{xii}$	62.476(10)
$Mn7^{xiv}—Mn7—Mn1^{xviii}$	64.473(12)	$Mn5^{xii}—Ge2—Mn4^{xii}$	62.582(12)
$Co1^{xii}—Mn7—Mn1^{xviii}$	101.025(15)	$Mn5^{xiii}—Ge2—Mn4^{xii}$	177.474(16)
$Co1^{xv}—Mn7—Mn1^{xviii}$	53.712(11)	$Mn4^{xiii}—Ge2—Mn4^{xii}$	116.459(18)
$Co4^{xiv}—Mn7—Mn1^{xviii}$	124.418(10)	$Co3^{xviii}—Ge3—Co3^{vii}$	180.000(13)
$Ge2—Mn7—Mn1^{xviii}$	103.377(13)	$Co3^{xviii}—Ge3—Co3^{x}$	116.771(13)
$M2^{xiv}—Mn7—Mn1^{xviii}$	94.683(11)	$Co3^{vii}—Ge3—Co3^{x}$	63.229(13)
$M2^{xvi}—Mn7—Mn1^{xviii}$	143.602(15)	$Co3^{xviii}—Ge3—Co3^{xx}$	63.229(13)
$Mn4^{xii}—Mn7—Mn1^{xviii}$	157.625(19)	$Co3^{vii}—Ge3—Co3^{xx}$	116.771(13)
$Mn4^{xiii}—Mn7—Mn1^{xviii}$	64.388(12)	$Co3^{x}—Ge3—Co3^{xx}$	180.000(13)
$Co1^{ix}—Co1—Ge2^{vii}$	122.215(7)	$Co3^{xviii}—Ge3—Co4^{xxi}$	117.046(9)
$Co1^{ix}—Co1—Co5^{vii}$	121.811(8)	$Co3^{vii}—Ge3—Co4^{xxi}$	62.954(9)
$Ge2^{vii}—Co1—Co5^{vii}$	109.809(13)	$Co3^{x}—Ge3—Co4^{xxi}$	62.954(9)
$Co1^{ix}—Co1—Ge1^{vii}$	121.709(7)	$Co3^{xx}—Ge3—Co4^{xxi}$	117.046(9)
$Ge2^{vii}—Co1—Ge1^{vii}$	104.593(12)	$Co3^{xviii}—Ge3—Co4$	62.955(9)
$Co5^{vii}—Co1—Ge1^{vii}$	60.572(12)	$Co3^{vii}—Ge3—Co4$	117.045(9)
$Co1^{ix}—Co1—Co6^{vii}$	120.463(7)	$Co3^{x}—Ge3—Co4$	117.045(9)
$Ge2^{vii}—Co1—Co6^{vii}$	57.907(12)	$Co3^{xx}—Ge3—Co4$	62.955(9)
$Co5^{vii}—Co1—Co6^{vii}$	107.775(13)	$Co4^{xxi}—Ge3—Co4$	180.000
$Ge1^{vii}—Co1—Co6^{vii}$	57.762(12)	$Co3^{xviii}—Ge3—Mn6^{vi}$	63.673(11)
$Co1^{ix}—Co1—Mn1^{i}$	63.716(7)	$Co3^{vii}—Ge3—Mn6^{vi}$	116.327(11)
$Ge2^{vii}—Co1—Mn1^{i}$	128.970(15)	$Co3^{x}—Ge3—Mn6^{vi}$	63.113(11)
$Co5^{vii}—Co1—Mn1^{i}$	64.089(11)	$Co3^{xx}—Ge3—Mn6^{vi}$	116.887(11)
$Ge1^{vii}—Co1—Mn1^{i}$	112.336(15)	$Co4^{xxi}—Ge3—Mn6^{vi}$	63.762(8)
$Co6^{vii}—Co1—Mn1^{i}$	170.093(15)	$Co4—Ge3—Mn6^{vi}$	116.238(8)
$Co1^{ix}—Co1—Co1^{xvii}$	180.000	$Co3^{xviii}—Ge3—Mn6^{xix}$	116.327(11)
$Ge2^{vii}—Co1—Co1^{xvii}$	57.787(7)	$Co3^{vii}—Ge3—Mn6^{xix}$	63.673(11)

Co5^{vii}—Co1—Co1^{xvii}	58.187(8)	Co3^{x}—Ge3—Mn6^{xix}	116.887(11)
Ge1^{vii}—Co1—Co1^{xvii}	58.289(7)	Co3^{xx}—Ge3—Mn6^{xix}	63.113(11)
Co6^{vii}—Co1—Co1^{xvii}	59.537(7)	Co4^{xxi}—Ge3—Mn6^{xix}	116.238(8)
Mn1^{i}—Co1—Co1^{xvii}	116.283(7)	Co4—Ge3—Mn6^{xix}	63.762(8)
Co1^{ix}—Co1—Mn7^{vii}	118.800(7)	Mn6^{vi}—Ge3—Mn6^{xix}	180.000(14)
Ge2^{vii}—Co1—Mn7^{vii}	65.142(12)	Co3^{xviii}—Ge3—Mn6^{vii}	116.887(11)
Co5^{vii}—Co1—Mn7^{vii}	61.299(13)	Co3^{vii}—Ge3—Mn6^{vii}	63.113(11)
Ge1^{vii}—Co1—Mn7^{vii}	110.976(13)	Co3^{x}—Ge3—Mn6^{vii}	116.327(11)
Co6^{vii}—Co1—Mn7^{vii}	112.585(13)	Co3^{xx}—Ge3—Mn6^{vii}	63.673(11)
Mn1^{i}—Co1—Mn7^{vii}	69.360(12)	Co4^{xxi}—Ge3—Mn6^{vii}	63.762(8)
Co1^{xvii}—Co1—Mn7^{vii}	61.202(7)	Co4—Ge3—Mn6^{vii}	116.238(8)
Co1^{ix}—Co1—Mn4	64.861(7)	Mn6^{vi}—Ge3—Mn6^{vii}	117.381(15)
Ge2^{vii}—Co1—Mn4	67.892(11)	Mn6^{xix}—Ge3—Mn6^{vii}	62.619(15)
Co5^{vii}—Co1—Mn4	120.371(16)	Co3^{xviii}—Ge3—Mn6^{xviii}	63.113(11)
Ge1^{vii}—Co1—Mn4	172.449(13)	Co3^{vii}—Ge3—Mn6^{xviii}	116.887(11)
Co6^{vii}—Co1—Mn4	116.423(16)	Co3^{x}—Ge3—Mn6^{xviii}	63.673(11)
Mn1^{i}—Co1—Mn4	73.406(15)	Co3^{xx}—Ge3—Mn6^{xviii}	116.327(11)
Co1^{xvii}—Co1—Mn4	115.141(7)	Co4^{xxi}—Ge3—Mn6^{xviii}	116.238(8)
Mn7^{vii}—Co1—Mn4	65.771(12)	Co4—Ge3—Mn6^{xviii}	63.762(8)
Co1^{ix}—Co1—Mn5	65.669(7)	Mn6^{vi}—Ge3—Mn6^{xviii}	62.619(15)
Ge2^{vii}—Co1—Mn5	65.758(11)	Mn6^{xix}—Ge3—Mn6^{xviii}	117.381(15)
Co5^{vii}—Co1—Mn5	171.438(13)	Mn6^{vii}—Ge3—Mn6^{xviii}	180.00(2)
Ge1^{vii}—Co1—Mn5	112.613(14)	Co3^{xviii}—Ge3—Mn3	115.47(1)
Co6^{vii}—Co1—Mn5	63.686(10)	Co3^{vii}—Ge3—Mn3	64.53(1)
Mn1^{i}—Co1—Mn5	124.467(12)	Co3^{x}—Ge3—Mn3	64.53(1)
Co1^{xvii}—Co1—Mn5	114.332(7)	Co3^{xx}—Ge3—Mn3	115.47(1)
Mn7^{vii}—Co1—Mn5	120.198(14)	Co4^{xxi}—Ge3—Mn3	117.398(12)
Mn4—Co1—Mn5	65.622(14)	Co4—Ge3—Mn3	62.602(12)
Co1^{ix}—Co1—Mn2^{i}	65.814(6)	Mn6^{vi}—Ge3—Mn3	116.482(8)
Ge2^{vii}—Co1—Mn2^{i}	169.331(13)	Mn6^{xix}—Ge3—Mn3	63.518(8)
Co5^{vii}—Co1—Mn2^{i}	66.959(12)	Mn6^{vii}—Ge3—Mn3	116.482(8)
Ge1^{vii}—Co1—Mn2^{i}	64.82(1)	Mn6^{xviii}—Ge3—Mn3	63.518(8)
Co6^{vii}—Co1—Mn2^{i}	112.608(15)	Co3^{xviii}—Ge3—Mn3^{xxi}	64.531(10)
Mn1^{i}—Co1—Mn2^{i}	59.755(14)	Co3^{vii}—Ge3—Mn3^{xxi}	115.469(10)

$Co1^{xvii}$—Co1—$Mn2^{i}$	114.183(6)	$Co3^{x}$—Ge3—$Mn3^{xxi}$	115.469(10)
$Mn7^{vii}$—Co1—$Mn2^{i}$	118.706(14)	$Co3^{xx}$—Ge3—$Mn3^{xxi}$	64.531(10)
Mn4—Co1—$Mn2^{i}$	122.711(12)	$Co4^{xxi}$—Ge3—$Mn3^{xxi}$	62.602(12)
Mn5—Co1—$Mn2^{i}$	115.954(13)	Co4—Ge3—$Mn3^{xxi}$	117.399(12)
$Co1^{ix}$—Co1—Mn3	66.028(7)	$Mn6^{vi}$—Ge3—$Mn3^{xxi}$	63.517(8)
$Ge2^{vii}$—Co1—Mn3	112.912(13)	$Mn6^{xix}$—Ge3—$Mn3^{xxi}$	116.483(8)
$Co5^{vii}$—Co1—Mn3	116.614(14)	$Mn6^{vii}$—Ge3—$Mn3^{xxi}$	63.518(8)
$Ge1^{vii}$—Co1—Mn3	64.847(10)	$Mn6^{xviii}$—Ge3—$Mn3^{xxi}$	116.482(8)
$Co6^{vii}$—Co1—Mn3	63.393(11)	Mn3—Ge3—$Mn3^{xxi}$	180.000
$Mn1^{i}$—Co1—Mn3	114.106(13)		

(i) -x, 1-y, -z; (ii) -x, 1-y, z; (iii) 0.5-x, 0.5+y, 0.5+z; (iv) 0.5-x, 0.5+y, -0.5+z; (v) 0.5-x, 0.5+y, 0.5-z; (vi) -0.5+x, 0.5-y, -0.5-z; (vii) -0.5+x, 0.5-y, 0.5-z; (viii) 1-x, 1-y, -z; (ix) x, y, -z; (x) -0.5+x, 0.5-y, -0.5+z; (xi) 1-x, 1-y, z; (xii) 0.5+x, 0.5-y, 0.5-z; (xiii) 0.5+x, 0.5-y, -0.5-z; (xiv) 1-x, -y, -z; (xv) 0.5+x, 0.5-y, -0.5+z; (xvi) 1-x, -y, z; (xvii) x, y, 1-z; (xviii) 0.5-x, -0.5+y, -0.5+z; (xix) 0.5-x, -0.5+y, 0.5+z; (xx) 0.5-x, -0.5+y, 0.5-z; (xxi) -x, -y, -z.
